# 3D Interconnected Honeycomb-Like Multifunctional Catalyst for Zn–Air Batteries

**DOI:** 10.1007/s40820-022-00959-6

**Published:** 2022-12-31

**Authors:** Tianxu Jin, Junli Nie, Mei Dong, Binling Chen, Jun Nie, Guiping Ma

**Affiliations:** 1 State Key Laboratory of Chemical Resource Engineering, Beijing University of Chemical Technology, Beijing, 100029 People’s Republic of China; 2 College of Engineering, Mathematics and Physical Science, University of Exeter, Exeter, EX4 4QF UK

**Keywords:** Fe/Co nanoparticles, Core–shell microspheres, Multifunctional catalyst, Stability

## Abstract

Developing high-performance and low-cost electrocatalysts is key to achieve the clean-energy target. Herein, a dual regulation method is proposed to prepare a 3D honeycomb-like carbon-based catalyst with stable Fe/Co co-dopants. Fe atoms are highly dispersed and fixed to the polymer microsphere, followed by a high-temperature decomposition, for the generation of carbon-based catalyst with a honeycomb-like structure. The as-prepared catalyst contains a large number of Fe/Co nanoparticles (Fe/Co NPs), providing the excellent catalytic activity and durability in oxygen reduction reaction, oxygen evolution reaction and hydrogen evolution reaction. The Zn-air battery assembled by the as-prepared catalyst as air cathode shows a good charge and discharge capacity, and it exhibits an ultra-long service life by maintaining a stable charge and discharge platform for a 311-h cycle. Further X-ray absorption fine structure characterization and density functional theory calculation confirms that the Fe doping optimizes the intermediate adsorption process and electron transfer of Co.

**Supplementary Information:**

The online version contains supplementary material available at 10.1007/s40820-022-00959-6.

## Highlights

- The unique initiator doping strategy is adopted to achieve high dispersion of Fe atoms in the catalyst.
- The 3D interconnected structure manufactured by morphology control exhibited excellent electrocatalytic performance in speeding up the electrolytes streaming and electron transfer.
- The Zn-air battery assembled with catalyst Fe_8_Co_0.2_-NC-800 could maintain a stable charge discharge platform in 1701 cycles of 311 h.

**Supplementary Information:**

The online version contains supplementary material available at 10.1007/s40820-022-00959-6.

## Graphical abstract

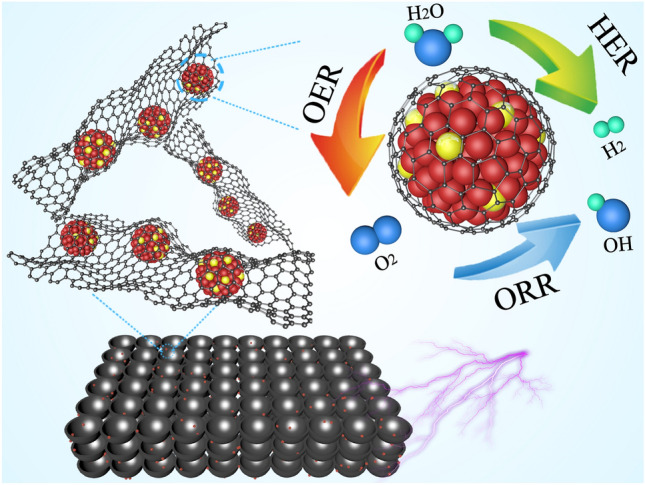

## Introduction

Zinc-air battery (ZAB) is considered as one of the promising green energy conversion devices because of its high theoretical energy density [1, 2], rich zinc reserves in the earth crust and the safety of nonflammable electrolyte [3–5]. However, the slow oxygen reduction reaction (ORR) and oxygen evolution reaction (OER) in the charge/discharge process limit its large-scale commercial application [6–8]. The most commonly used cathode electrocatalysts are noble-metal-based compounds such as Pt-based catalysts, but they bring disadvantages of poor chemical stability, environmental damage and high cost [9, 10]. In order to solve these problems, designing noble-metal-free catalysts is an effective strategy [11]. Among them, transition metal-nitrogen-carbon materials exhibit high ORR and OER activities due to their synergistic effects between metallic particles and carbon atoms with various doped atoms, making rich local electronic structures and abundant active sites [12].

A high number of catalytical active sites and their sufficient exposure to electrolytes are important for the construction of high-efficient electrocatalysts [13]. Take an example of the single-atom-catalyst, a sufficient exposure of active sites on the material surface or under the carbon layer resulted in extremely high electrocatalytic performance [14–17]. However, the surface energy of catalytic active sites is so high that active sites are very easy to be agglomerated, resulting in low stability. Numerous studies have shown that morphology regulation can effectively construct electrocatalytic materials with large specific surface area microstructures, such as hollow nanotube structured materials with remarkable bifunctional oxygen electrocatalytic properties prepared by self-template synthesis method [18]. Among these different structures, hollow core–shell sphere structure can provide high specific surface area and load more active sites [19], for example, using carbon layer as a substrate to fix the nanoparticles (NPs) or sub nanostructures has been widely reported [20–23]. Such hollow structure is helpful to stabilize the active sites and accelerate the mass transfer process in the electrochemical process [13]. Conductivity is also one of the performance sources of energy devices [24]. The asymmetry and electronegativity of electron spin density caused by carbon as the matrix, and other atom dopants can change the local electron cloud density, therefore further improve the ORR performance [25, 26].

In this work, we synthesized a three-dimensional interconnected honeycomb-like electrocatalyst, which is derived from hollow carbon microspheres by the pyrolysis of core–shell microspheres. During the synthesis, polystyrene (PS) microspheres were used as hard template, polyaniline (PANI) layer was grown *in-situ* on their surface as the precursor of carbon matrix. Instead of using the traditional ammonium persulfate initiator, FeCl_3_ was used as oxidant to initiate aniline polymerization. Fe^3+^ ions interact with -NH- on the polyaniline chain, so that Fe atoms can be doped into the carbon matrix and dispersed efficiently. This is beneficial to the formation of Fe doped Co nanoparticles (Fe/Co NPs). In terms of the pyrolysis, melamine was mixed with as-prepared precursors to form a gas atmosphere with a high nitrogen content. PS microspheres had an etching effect on the carbon matrix, as they decomposed rapidly at temperature of 450 °C. After pyrolysis, the Fe_8_Co_0.2_-NC-800 catalyst was generated with a three-dimensional interconnected honeycomb-like structure. Combined with X-ray diffraction (XRD), X-ray absorption fine structure (XAFS) and other characterization, the metal NPs in the Fe_8_Co_0.2_-NC-800 mainly exposed the (111) crystal plane of Co, and there was no crystal form of Fe species, and the characterization results of EXAFS confirmed the presence of Fe-Co coordination in Fe/Co NPs, where Fe/Co NPs with high catalytic activity were formed. This means that the high dispersion of Fe atoms in Co NPs further activated the multi-functional catalytic activity of Co NPs. Specifically, the half wave potential of the optimal sample Fe_8_Co_0.2_-NC-800 reached 0.820 V in the linear sweep voltammetry (LSV) test of ORR. In the LSV test of OER, the overpotential at 10 mA cm^−2^ was 0.402 V. In the HER test in alkaline electrolyte, the overpotential is 0.291 V at 10 mA cm^−2^. After a series of electrocatalytic evaluations and Zn-air battery tests, Fe_8_Co_0.2_-NC-800 was confirmed to exhibit a high electrocatalytic performance, which is comparable to the benchmark Pt/C. In particular, the battery can maintain a good charging and discharging platform and excellent stability in the 311-h long test cycle charging and discharging test. Density functional theory (DFT) results show that the (111) crystal surface exposed by Fe/Co NPs is the main source of catalytic activity, and the synergistic effect of Fe and Co atoms is the key for the high electrocatalytic activity.

## Experiments Sections

### Preparation of PS Microspheres

In a typical process. 1 g of polyvinylpyrrolidone (PVP) and 80 mL of deionized water was added to a four-necked flask and stirred thoroughly. After PVP was dissolved, 11 mL of styrene (St) was added in, with continuous access to N_2_ during the period. 20 mL of an aqueous solution with 0.35 g of potassium persulfate (KPS) was added, stirred evenly, and raised the temperature to 70 °C for 24 h.

### Preparation of Fe_x_Co_y_-PS/PANI Core–shell Microspheres

30 mL of deionized water was first added to the beaker, and emulsion containing 0.2 g of PS microspheres was then added in. After sonicating to homogeneity, 1 mmol aniline (An) was added under magnetic stirring. A certain amount of FeCl_3_·6H_2_O was dissolved in 20 mL H_2_O (the amounts of FeCl_3_·6H_2_O are 1, 4, 8, and 12 mmol, respectively), then was quickly added to the above system, magnetic stirred at room temperature for 8 h. After the reaction was completed, samples were collected by centrifugation at 9,000 rpm. A certain amount of Co(NO_3_)_2_·6H_2_O was dissolved in 5 mL H_2_O (the amounts of Co(NO_3_)_2_·6H_2_O are respectively 0.2, 0.4, 0.6, and 0.8 mmol), and was mixed with Fe_8_-PS/PANI under ultrasonication with the Co(NO_3_)_2_·6H_2_O solution thoroughly. The resulting mixture was quickly frozen with liquid nitrogen and then put into a freeze dryer for 2 days to freeze-dry. The obtained powder was named as Fe_x_Co_y_-PS/PANI.

### Preparation of Fe_8_Co_0.2_-NC-T

The Fe_8_Co_0.2_-PS/PANI sample was thoroughly mixed and ground with melamine. The mass ratio of Fe_8_Co_0.2_-PS/PANI to melamine was 1:10. Under Ar atmosphere, the temperature was raised to the target temperature at a rate of 5 °C min^−1^. It was carbonized for 2 h at 700, 800, and 900 ℃, and the obtained catalyst was named as Fe_8_Co_0.2_-NC-T, where T stands for carbonization temperature. For comparison, both Fe_8_-NC-T and Co_0.2_-NC-T were also prepared by using the above method.

### Assembly and Test of Aqueous Zn-Air Batteries

In our previous work, 19 mg of catalyst and 9.8 mg of conductive carbon black were ultrasonic dispersed in a mixed solution of 3 mL of ethanol and 0.22 mL of Nafion for 1 h. The mass load of catalyst in the prepared ink was 0.84 mg cm^−3^. In terms of the battery cathode preparation, 100 μL catalyst ink droplets dropped onto the hydrophobic carbon paper was used as gas diffusion layer, the formation of gas diffusion diameter was 10 mm, and a layer of composite nickel foam was used as a fluid collector. The anode adopts 1 mm thick zinc sheet, which was polished before use. A mixture of 6 M K(OH) and 0.2 M Zn(CH_3_COO)_2_ was selected as the electrolyte. As a comparison, Pt + RuO_2_ was prepared under the same conditions as the control group. CHI760E (Shanghai Chenhua) was used to measure the open circuit voltage and LSV polarization curve, and LAND-CT2001A was used for long-term discharge curve test and long-term charge discharge test with a cycle of 10 min (i.e., discharge for 5 min and charge for 5 min). The test current density on LAND-CT2001A is 5 mA cm^−2^.

### Assembly and Test of Flexible Zn-Air Batteries

In a typical process, 6 g of acrylamide was dissolved in 6 g of deionized water, stirred and dissolved, then 6 mg of N, N'- methylebis(acrylamide) was added for stirring and dissolution, and 60 mg of 2-hydroxy-4'-(2-hydroxythoxy)-2-methyl-propophe was added during stirring, followed by ultrasonication for 10 min. The mixture was injected into a silica gel mold with a length of 5 cm, a width of 1 cm and a thickness of 2 mm, and cured with ultraviolet light with a wavelength of 365 nm for 15 min. The polyacrylamide (PAM) gel was dried at 60 °C in oven and soaked in 6 M K(OH) water and glycerin mixture for 3 days. The anode adopts zinc foil with a thickness of 0.08 mm, which was polished before use. The equation of gas diffusion layer is the same as that of aqueous Zn-Air batteries. Only hydrophobic carbon paper was replaced by hydrophilic carbon cloth. The test method is the same with that of aqueous Zn-Air batteries.

### Materials Characterizations

The morphology and structure of each sample were characterized by transmission electron microscope (TEM, FEI TECNAI G2 F20) under 200 kV accelerating voltage and scanning electron microscope (SEM, Hitachi s4700) under 20 kV accelerating voltage. HR-TEM images were obtained by FEI TECNAI G2 F20. EDS mapping images were collected by JEM-2100F 200 kV. X-ray diffraction (XRD) pattern was used by X-ray diffractometer (35 kV, 200 mA) equipped with a Cu Kα radiation system to analyze the crystal phase information of the radiation system. Nicolet-is5 infrared spectrometer was used to collect Fourier transform infrared spectrum information. Micromeritics ASAP 2020 was used for BET test to obtain N_2_ adsorption/desorption curve. X-ray photoelectron spectroscopy (XPS) was performed by Thermo Fisher Scientific (ESCALAB 250) to analyze the valence states of various elements in samples. Raman spectra were measured by Raman spectrometer (Invia Reflex, Renishaw).

### Electrochemical Measurements

All electrochemical experiments were carried out at room temperature using CHI 760E (CHI Instrument) and standard three electrodes configured with rotating ring disk electrode. The solution environment used is 0.1 M KOH (potassium hydroxide) solution and 1 M KOH solution. The working electrode is a rotating ring-disk electrode (i.e., 4 mm in diameter), and the reference electrode and counter electrode are Ag/AgCl and graphite rod respectively. Before the test, the glassy carbon electrode was polished with 50 nm Al_2_O_3_ powder, and then cleaned with ethanol and deionized water to provide a mirror. Then, 5 mg of catalyst was dispersed in the mixed solution of 1 mL ethanol and 100 µL Nafion solution, and was under ultrasonication for 30 min to prepare catalyst ink. 5 µL of catalyst ink was transferred to glassy carbon electrode and was dried at room temperature for all electrochemical tests.

For ORR test, before CV test, purge O_2_ or N_2_ into 0.1 M KOH solution for 30 min to achieve gas saturation, and scan CV curve in the range of 0.1 to 1.1 V (vs. RHE) at a scanning rate of 50 mV s^−1^. LSV curve measurement was carried out in 0.1 M KOH saturated with O_2_ at room temperature. The scanning rate was 5 mV s^−1^ at different speeds of 625 ~ 2500 rpm. According to Koutecky-Levich equation, the electron transfer number (n) and dynamic current density (*J*_K_) can be calculated:

1 $$\frac{1}{J} = \frac{1}{{J_{K} }} + \frac{1}{{J_{L} }} = \frac{1}{{J_{K} }} + \frac{1}{{B\omega^{0.5} }}$$

2 $$B = 0.62nFC_{0} D_{0}^{{{\raise0.7ex\hbox{$2$} \!\mathord{\left/ {\vphantom {2 3}}\right.\kern-\nulldelimiterspace} \!\lower0.7ex\hbox{$3$}}}} V^{{{\raise0.7ex\hbox{${ - 1}$} \!\mathord{\left/ {\vphantom {{ - 1} 6}}\right.\kern-\nulldelimiterspace} \!\lower0.7ex\hbox{$6$}}}}$$

In Eq. (1), *J* (mA cm^−2^) is the measured disc current density, *J*_K_ is the dynamic limit current, ω is the angular velocity of electrode rotation, and B is the slope of the K-L diagram. In Eq. (2), F is the Faraday constant (96,485C mol^−1^), *C*_0_ is the oxygen concentration in the electrolyte (1.2 × 10^–6^ mol cm^−3^), *D*_0_ is the oxygen diffusion coefficient in 0.1 M KOH (1.9 × 10^–5^ cm^2^ s^−1^), *V* is the kinematic viscosity of the electrolyte (0.01 cm^2^ s^−1^), and n represents the number of electron transfer per oxygen molecule.

The long-term stability evaluation of ORR test catalyst was carried out at 0.7 V (vs. RHE) voltage and 1,600 rpm. After 1,000 CV scans, LSV scans were performed again to test the stability of the catalyst.

For the OER test, the CV curve was carried out in 1 M KOH solution at a sweep rate of 50 mV s^−1^ in the range of 1.0 ~ 2.1 V (vs. RHE). In the same voltage range, the LSV curve of OER was acquired with IR corrected. For HER, after purging N_2_ into 1 M KOH solution for 30 min, CV curve scanning was carried out with a scanning speed of 50 mV s^−1^ in the range of—0.7 ~ 0 V (vs. RHE), and LSV test was carried out in the same voltage range.

## Results and Discussion

### Physical Characterizations

**Fig. 1:**
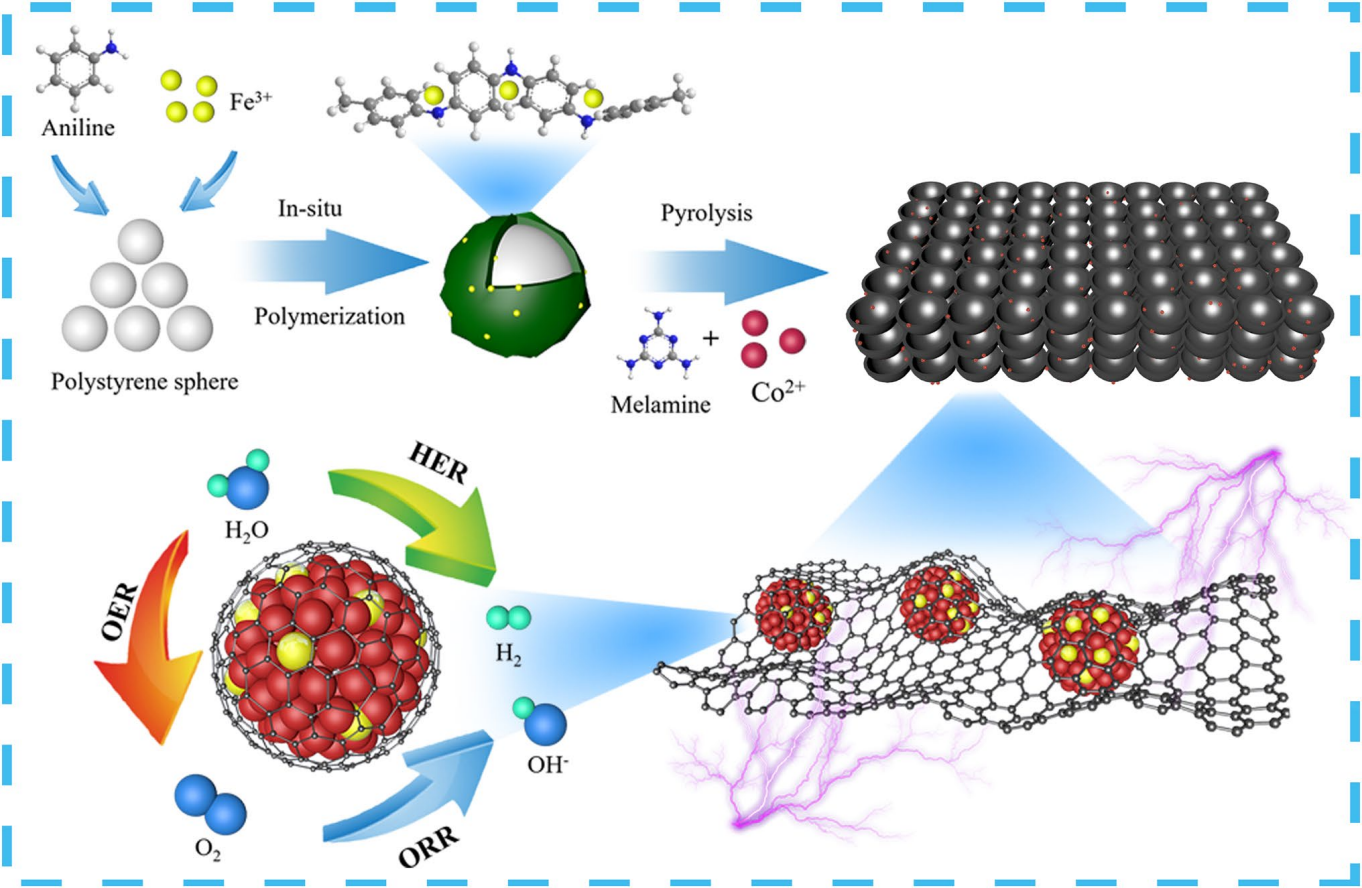
Schematic illustration of the fabrication of Fe_8_Co_0.2_-NC-800 catalyst

**Fig. 2:**
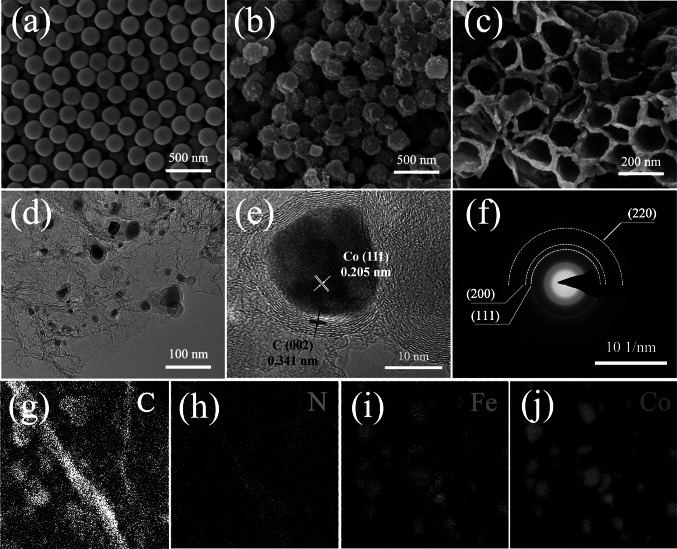
SEM images of **a** PS, **b** Fe_8_-PS / PANI, **c** Fe_8_Co_0.2_-NC-800; **d** TEM, **e** HR-TEM images, **f** SAED pattern and **g-j** corresponding elemental mapping images of Fe_8_Co_0.2_-NC-800

Our three-dimensional interconnection honeycomb-like structured material was prepared by the hard template method. PANI layer was first polymerized *in-situ* on the surface of the PS microspheres template to form PS/PANI microspheres. Melamine, which is beneficial to the N doping, was then mixed with PS/PANI microspheres before carbonization (Fig. 1). As shown in Fig. 2a, the as-prepared PS microspheres have a smooth surface with an average size of 200 ± 10 nm. The C = O stretching vibration peak and C-N stretching vibration peak can be observed at 1670 cm^−1^ in the Polyvinylpyrrolidone (PVP) covering sample (Fig. S1a). After that, with the aid of FeCl_3_·6H_2_O, the PANI monomer was polymerized rapidly, forming a layer of coating on the surface of PS microspheres. In the meanwhile, Fe^3+^ was also incorporated into the PANI layer. The as-prepared PS/PANI microspheres show a regular spherical shape with some adhesion between each spheres (Fig. 2b). The benzene ring of 1456 cm^−1^ and the stretching vibration peak of N = C at 1160 cm^−1^ both confirm the successful formation of PANI (Fig. S1b) [25]. The morphology of PS/PANI samples can be modulated by adjusting the amount of FeCl_3_·6H_2_O. With the increase of FeCl_3_·6H_2_O, the shape of PANI gradually turned to be fibrous, and the adhesion between spheres gradually intensified due to the fast polymerization rate (Fig. S2b) [27]. After further addition of Co and carbonization at 800 ℃ for 2 h, the PANI layer turned into a carbon shell with Fe, Co, and N-dopants. As PS decomposed at about 450 ℃, the generated gas punched and broke through the PANI shell to form a bowl-like structure. In addition, as melamine decomposed violently at 320 ℃, it generated a gas atmosphere containing N during carbonization, further contributing N-dopant to the sample (Fig. S3). As a result, due to the appropriate design of PS/PANI microspheres, the finally formed carbon-based materials show an interconnected honeycomb-like structure (Fig. 2c).

TEM image (Fig. 2d) clearly shows that Fe/Co NPs are dispersed in the honeycomb-like carbon matrix. HR-TEM image (Fig. 2e) shows that the size of Fe/Co NPs is around 20 nm. Its lattice spacing is 0.205 nm, which is assigned to the (111) planes of Co crystal [28–30], while its carbon layer covering has the characteristics of the C (002) planes in the lattice spacing of 0.341 nm [31]. The selected area diffraction (SAED) (Fig. 2f) has a clear diffraction pattern, and its concentric rings can be attributed to the (111), (200), (220) crystal planes of Co crystal from inside to outside [28]. Element mapping images (Fig. 2g-j) show the dispersion of C, N, Fe, and Co elements through the as-prepared material, and their corresponding content can be detected (Fig. S4). The content of Co in carbon matrix is 6.3 wt% and the content of Fe is 4.6 wt% detected by ICP-MS analysis (Table S1). There is a relatively concentrated distribution of Fe and Co elements in Fe/Co NPs, suggesting the formation of Fe/Co NPs. As a result, combined with HR-TEM and element analysis, it is speculated that the Fe/Co NPs is a co-crystal. The doping of Fe atoms could regulate the local electron cloud density distribution of Co NPs, so that to optimize the catalytic performance of the material [32].

**Fig. 3:**
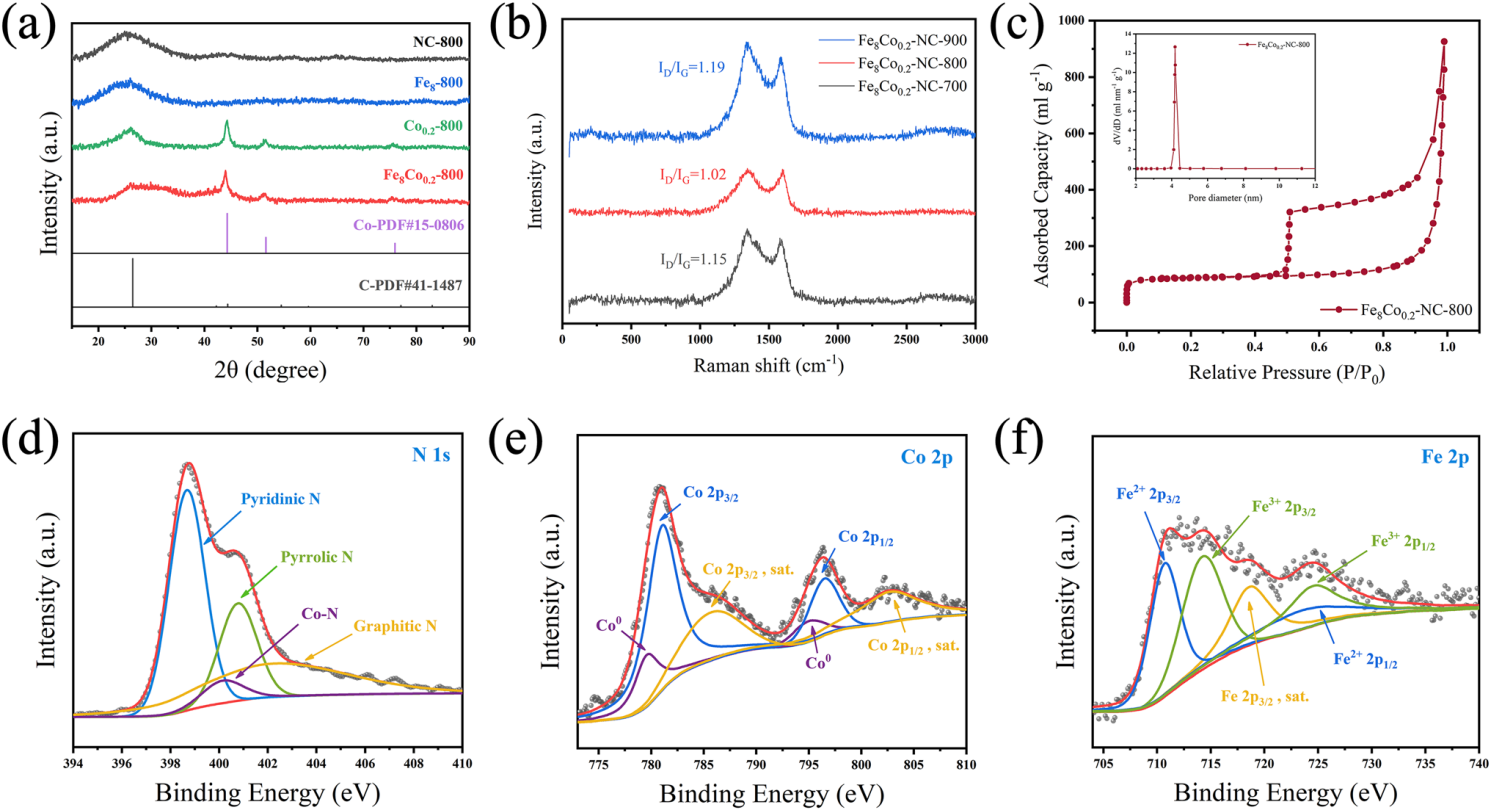
**a** XRD patterns, **b** Raman spectra of Fe_8_Co_0.2_-NC-800 and **c** Nitrogen adsorption–desorption isotherm of Fe_8_Co_0.2_-NC-800 (insert shows pores size distribution); **d** N 1*s*, **e** Co 2*p*, **f** Fe 2*p* high resolution XPS spectrum of Fe_8_Co_0.2_-NC-800

XRD patterns (Fig. 3a) confirm the presence of carbon and Fe/Co NPs phase. The wide peaks at 26.38° and 42.22° in Fe_8_Co_0.2_-NC-800 are attributed to the (002) and (100) crystal planes of graphite carbon (JCPDS No. 41–1487), respectively [33–36]. The reference samples Co_0.2_-NC-800, Fe_8_-NC-800, and NC-800 can also find the presence of graphite carbon. No crystalline diffraction peaks of Fe and its compounds can be identified [32]. This XRD result infers that Fe species maintained a highly dispersed state at high temperature, such as single atom or cluster, because they were anchored by polyaniline molecular chains during the preparation process [22, 36]. The peaks at 44.22°, 51.52°, and 75.85° in Fe_8_Co_0.2_-NC-800 and Co_0.2_-NC-800 samples show the presence of Co metal species (JCPDS No. 15–0806), but no FeCo alloy phase can be identified. Therefore, it is proposed that Fe was highly dispersed in Co NPs to form Fe/Co NPs, which is consistent with the results of HR-TEM (Fig. 2e). The Raman spectra (Fig. 3b) show that the ratio of D (1350 cm^−1^) strength to G (1580 cm^−1^) (*I*_D_/*I*_G_) of Fe_8_Co_0.2_-NC-800 is 1.02, indicating that it has a high degree of graphitization, along with some defects in the carbon matrix. However, in terms of Fe_8_Co_0.2_-NC-700 and Fe_8_Co_0.2_-NC-900, the defect concentrations are so high that it could lead to the reduction of *sp*^2^ carbon atoms and the decrease of conductivity, further reducing their electrocatalytic performance [8, 25].

The surface element composition and element state of Fe_8_Co_0.2_-NC-800 were determined by XPS. The peaks at 398.68 and 400.83 eV shown in the enlarged figure of N 1*s* (Fig. 3d) are mainly attributed to pyridine-N and pyrrole-N, while the weak peaks at 400.23 and 402.43 eV belong to Co–N and graphite-N, respectively [37]. The weak intensity of Co–N peak suggests the N and Co coordination at the junction of Co NPs and graphite carbon layer, which is conducive to ORR catalytic activity. Pyridine-N and graphite-N are generally considered to be one of the key active sites of ORR, but the mechanism has not been determined yet [38]. Compared with the N 1*s* enlarged view of Fe_8_-NC-800 and Co_0.2_-NC-800 (Fig. S6), Fe_8_Co_0.2_-NC-800 contains a higher content of pyridine-N, which is beneficial to the improvement of catalyst performance [36, 39]. The N 1*s* enlarged diagram of NC-800 sample (Fig. S7) shows that there are stronger pyrrole-N peaks rather than that in Fe_8_Co_0.2_-NC-800, which means that the addition of metal elements is no doubt conducive to the formation of pyridine-N [13]. The metal Co at 779.73 and 795.25 eV can be identified in Fe_8_Co_0.2_-NC-800 (Fig. 3e), which is attributed to the Co NPs, while the valence states of Co 2*p*_3/2_ at 781.13 eV and Co 2*p*_1/2_ at 796.53 eV are mainly attributed to the complex compounds produced after carbonization, including Fe-Co, Co–N and Co–C [37, 40]. The peaks in the enlarged view of Fe 2*p* (Fig. 3f) can be divided into two main groups. The peaks of 710.78 and 724.68 eV are attributed to Fe^2+^ 2*p*_3/2_ and Fe^2+^ 2*p*_1/2_, while 714.33 and 724.83 eV are attributed to Fe^3+^ 2*p*_3/2_ and Fe^3+^ 2*p*_1/2_, respectively [41]. It suggests that Fe forms a complex coordination structure with C and N elements. Because of the very small size/content of Fe, it is reasonable that the diffraction peak cannot be identified from the XRD patterns (Fig. 3a). In the Fe 2*p* enlarged spectrum (Fig. S8) of Fe_8_-NC-800, the obvious differences between Fe_8_Co_0.2_-NC-800 are the Fe^2+^ 2*p*_3/2_ and the shift of Fe^3+^ 2*p*_1/2_, of which results are consistent with those of EDS mapping (Fig. 2g-j) and XRD patterns (Fig. 3a).

**Fig. 4:**
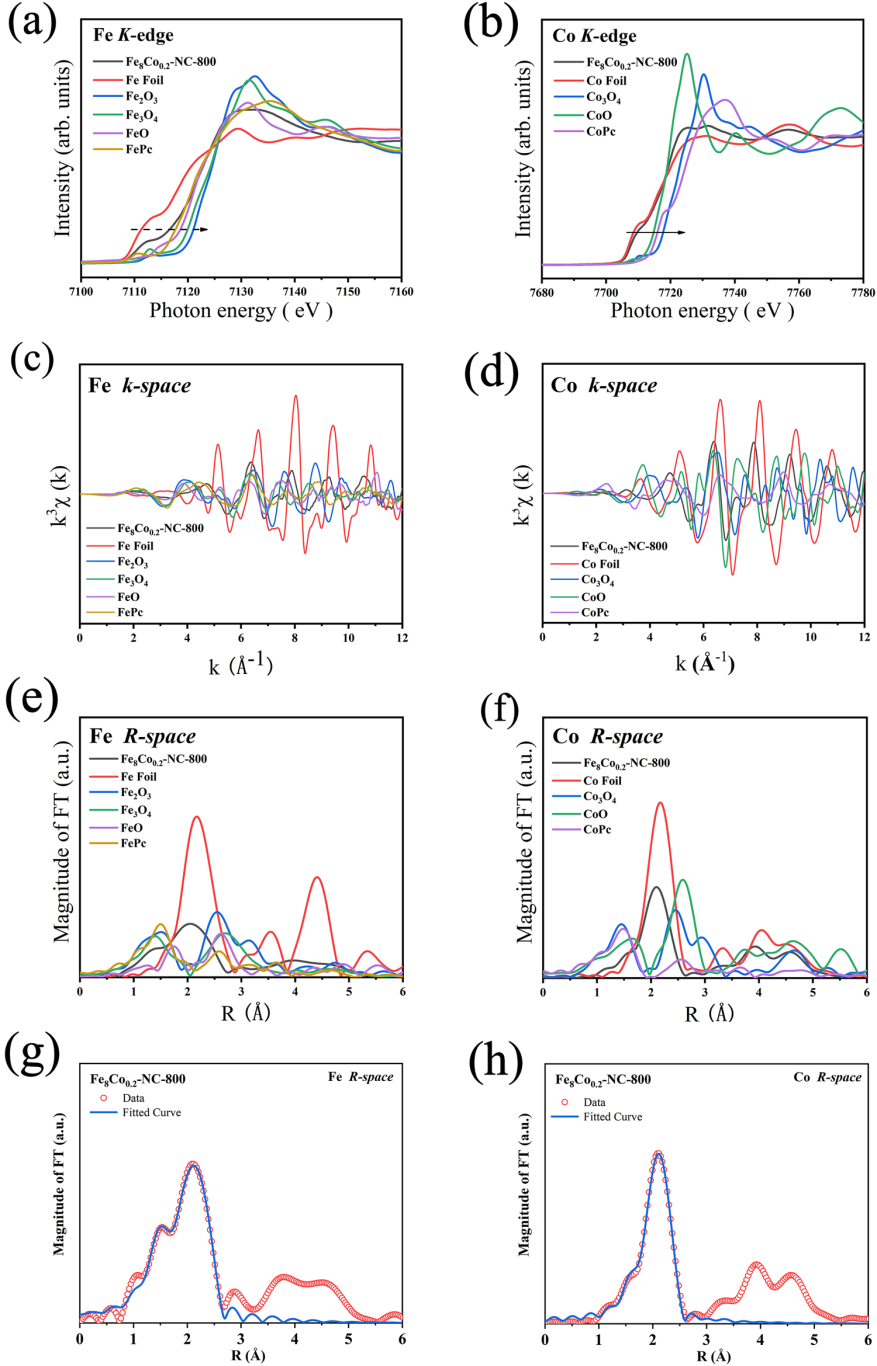
Normalized XANES spectra of samples at **a** Fe K-edge, **b** Co K-edge; Fourier transform (FT) EXAFS of **c** Fe K-edge, **d** Co K-edge in k-space and **e** Fe K-edge, **f** Co K-edge in R-space; The corresponding EXAFS fitting curve of **g** Fe and **h** Co in R-space

In order to verify the active sites, specific coordination structures and electron cloud distribution of Fe/Co NPs, XAFS test were carried out. As seem from the X-ray absorption near edge structure (XANES) of Fe (Fig. 4a), the position of the absorption edge in the arrow direction confirms the oxidation state of Fe. In Fe_8_Co_0.2_-NC-800, the Fe absorption edge is between Fe foil (0) and FeO (+ 2), indicating that the valence state of Fe in the sample is between 0 ~  + 2. In the XANES diagram of Co (Fig. 4b), the Co absorption edge is between Co foil (0) and CoO (+ 2), indicating that the valence state of Co in the sample is between 0 ~  + 2 valence and very close to 0 valence [36]. In the k-space diagram of Fourier transform extended X-ray absorption fine structure (EXAFS) (Fig. 4c-d), compared with Fe, Co element standard sample (Figs. S9-S10), there is no obvious strong amplitude signal in the high-k region after 6 Å, indicating that no heavy metal elements in Fe_8_Co_0.2_-NC-800 scattered with Co and Fe. This means no heavy metals are in the local region of the Co and Fe centers. In the Fe R-space diagram of EXAFS (Figs. 4e and S11d-f), it can be seen that the first shell and the second shell are connected together. This is because the distance between Fe–N bond and Fe-Co bond is very close with a value of 0.5 Å. However, the first shell of Fe–N can be clearly identified. The Fe-Co bond of the second shell is very close to the Fe foil bond length, and the bond length is very short. This result suggests the formation of alloy Fe-Co bonds [22]. Similarly, in Co R-space (Figs. 4f and S11a-c), the first shell and the second shell are connected together, and the bond length of the second shell is also compressed, confirming that Co and Fe are formed to be an alloy. Combined with XRD results (Fig. 3a), it can be verified that in the Fe/Co NPs, Fe atoms were highly dispersed in Co, and the Fe–N sites are presented [36, 41]. The EXAFS data of Fe_8_Co_0.2_-NC-800 at the Fe (Fig. 4g) and Co (Fig. 4h) k edges both fit well, confirming that the data in Table S1 is reliable. Through the comparison between Fe_8_Co_0.2_-NC-800 and the standard sample, Fe/Co in the Fe_8_Co_0.2_-NC-800 also shows as a form of alloy and the coordination with N. The coordination number of Co with N, Co with Fe/Co, Fe with N, Fe with Co/Fe is 3, 6, 3, and 5, respectively. Wavelet transform extended X-ray absorption fine structure (WT-EXAFS) data can identify the coordination information of samples [42]. From WT-EXAFS (Fig. S12) of Co in Fe_8_Co_0.2_-NC-800, it can be found that the peak is longer than that of Co foil, and some peaks are below 2 Å. These peaks are the signals of co Fe alloy. In addition, the bond lengths of Co–N and Co-Fe/Co are close, thus only one peak is displayed in R-space. In terms of Fe in Fe_8_Co_0.2_-NC-800 (Fig. S13), it can be found that a trailing peak is shifted to 1.5 Å, which is the signal of Fe–N bond. It also can be seen that the bond length of Fe/Co alloy is lower than that of Fe foil at about 2 Å [43].

### Electrochemical Tests

**Fig. 5:**
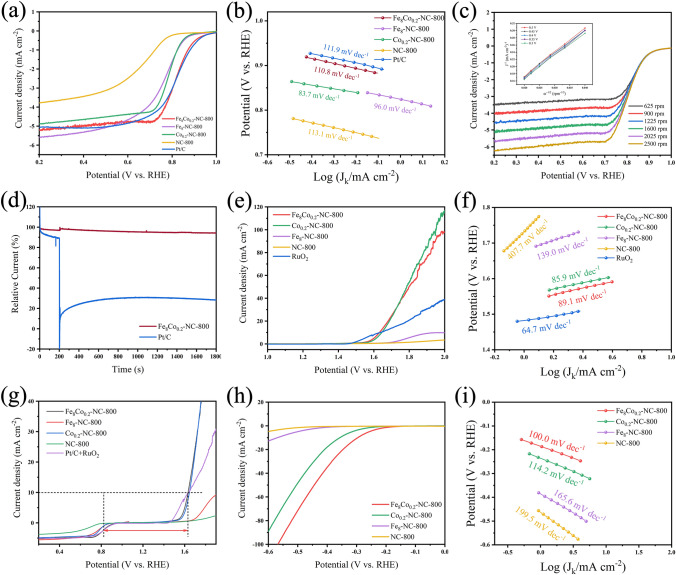
**a** LSV curves of Fe_8_Co_0.2_-NC-800, Fe_8_-NC-800, Co_0.2_-NC-800, NC-800, Pt/C and their **b** Tafel plots. **c** LSV curves at different speeds of Fe_8_Co_0.2_-NC-800 (insert shows K-L curve). **d** Methanol tolerance test of Fe_8_Co_0.2_-NC-800; **e** LSV curves of OER test and their **f** Tafel plots; **g** As-prepared catalysts for ORR/OER catalytic activity; **h** LSV curves of HER test and their **i** Tafel plots

Rotating ring disk electrode (RDE) was used to evaluate the ORR, OER, and HER performance of the prepared samples. In terms of the ORR optimization experiment, Fe-NC-800 catalysts with different Fe^3+^ and aniline ratios were all tested. Fe_8_-NC-800 was found to have the best performance, as it has a favorable morphology (Fig. S14). In the further experiment of Fe/Co NPs, it was found that the Fe_8_/Co_0.2_ ratio is optimal to the catalytic performance Fe/Co NPs (Fig. S15). In addition, in the optimization experiment of carbonization temperature, the temperature at 800 °C was found to be optimal (Fig. S16). This is because the carbon obtained at 800 °C has more appropriate defect concentration and conductivity, as shown in the result of Raman spectroscopy (Fig. 3b). As shown in Fig. 5a, Fe_8_Co_0.2_-NC-800 exhibited a superior ORR performance to the other reference samples, where its half wave potential (0.820 V) is equivalent to that of Pt/C catalyst. Its Tafel slope of 110.8 mV dec^−1^ is very close to that of Pt/C catalyst (Fig. 5b), indicating a fast ORR kinetics [27]. The linear volt ampere curves of at different speeds and the corresponding K-L curve show the high stability of Fe_8_Co_0.2_-NC-800 during the catalytic process (Fig. 5c), where the diffusion limit current of Fe_8_Co_0.2_-NC-800 increases steadily with the increase of rotating speed, but other reference samples exhibited inferior performances (Figs. S17-S18). In the stability test, Fe_8_Co_0.2_-NC-800 showed excellent stability after 1000 cyclic voltammetry scans, where its half wave potential was only attenuated by 4 mV (Fig. S19a). Because the microstructure of the catalyst may change after a long time of use, SEM and HRTEM were used to characterize the morphology of the catalyst before and after use. The SEM results show that the carbon matrix of the catalyst had some collapse and adhesion after use (Fig. S20a-b). The HRTEM (Fig. S20c-d) images of Fe/Co nanoparticles in the catalyst show that the structure of nanoparticles had almost no change before and after scanning, which proves that the slight attenuation of catalyst performance after use may be due to the change of carbon matrix. Such excellent stability of Fe/Co nanoparticles makes the high durability of the catalyst. After a 36,000 s chronoamperometric test (Fig. S19b), the current of Fe_8_Co_0.2_-NC-800 remained 87% of the original, which is higher than that of commercial Pt/C (i.e., 79.3%). Fe_8_Co_0.2_-NC-800 also exhibited an excellent methanol tolerance (Fig. 5d), the current of Fe_8_Co_0.2_-NC-800 was barely changed within 1,800 s even after the addition of methanol in 200 s. This durability and methanol tolerance can be attributed to the inertia of Fe/Co NPs to methanol, as well as the protection of carbon layer deposited on the surface of Fe/Co NPs (Fig. 2e) [27, 40, 44].

In addition to the excellent ORR performance of Fe_8_Co_0.2_-NC-800, a comprehensive study on its OER and HER performance was also carried out. As seen in Fig. 5e, Fe_8_Co_0.2_-NC-800 has an over potential of 1.632 V equivalent to the benchmark RuO_2_ at the current density of 10 mA cm^−2^. The electrochemical impedance spectroscopy (EIS) results show that Fe_8_Co_0.2_-NC-800 has the lowest charge transfer resistance among all the reference samples (Fig. S21). It means that Fe_8_Co_0.2_-NC-800 has the highest charge transfer efficiency. The Tafel slopes shown in Fig. 5f also support the above results. The voltage difference between OER at 10 mA cm^−2^ and the half wave potential of ORR (ΔE) is the standard for the bifunctional catalytic performance of a reaction catalyst. Among all the prepared catalysts, Fe_8_Co_0.2_-NC-800 has a voltage difference of 0.812 V, which is equivalent to that of Pt/C + RuO_2_, while it is superior to all the other catalysts (Figs. 5g and S22). The HER LSV results shown in Fig. 5h suggest that Fe_8_Co_0.2_-NC-800 still has a relevant high HER catalytic activity in alkaline electrolyte. The overpotential of Fe_8_Co_0.2_-NC-800 at 10 mA cm^−2^ is only 291 mV, and it has the lowest Tafel slope vale of 100.0 mV dec^−1^ (Fig. 5i), which is significantly better than Co_0.2_-NC-800, which proved that the incorporation of Fe effectively improved the catalytic performance of Co NPs. Compared with the same type of electrocatalyst reported in relevant literature, Fe_8_Co_0.2_-NC-800 not only has three electrocatalytic functions of ORR, OER and HER, but also has better performance than other catalysts (Table S6).

### Zn-Air Battery Test

**Fig. 6:**
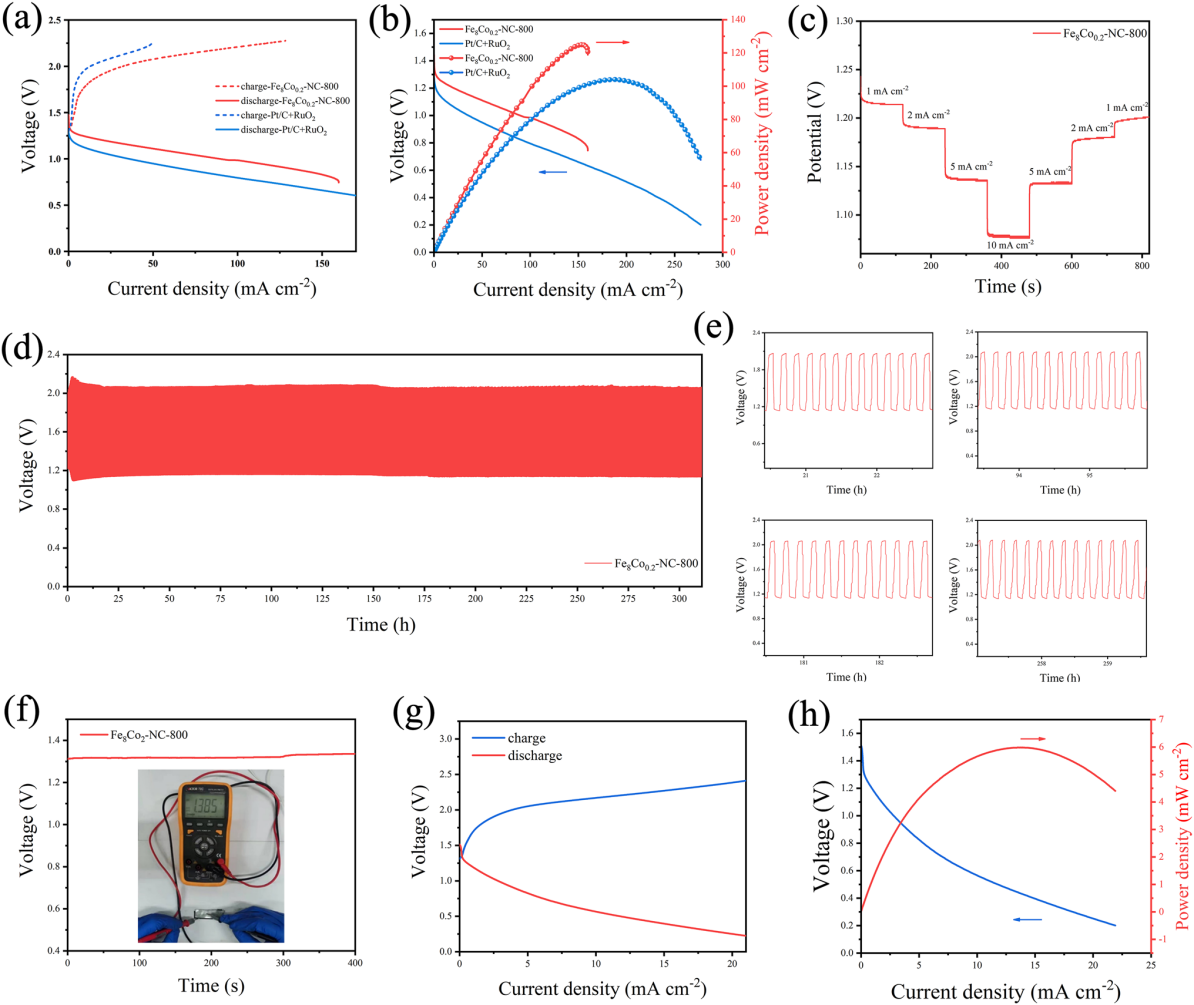
**a** Polarization curves of charge and discharge for Fe_8_Co_0.2_-NC-800 and the Pt/C + RuO_2_ and their **b** catalyst power density plots. **c** Rate capabilities of Fe_8_Co_0.2_-NC-800. **d** Constant current charge–discharge curves and **e** plots in on random time periods of ZAB with the Fe_8_Co_0.2_-NC-800 cathode. **f** The single flexible ZAB open-circuit voltage of using Fe_8_Co_0.2_-NC-800 as an air cathode (insert is digital photo of ZAB open circuit voltage test) and its **g** polarization curves of charge and discharge; **h** catalyst power density plots

The excellent ORR/OER catalytic activity of Fe_8_Co_0.2_-NC-800 make it possible to be as an air cathode for aqueous rechargeable ZAB. The assembled ZAB has an open circuit voltage of 1.426 V and can remain stable within 400 s (Fig. S23). Compared with the commercial catalyst 20 wt% Pt/C + RuO_2_, Fe_8_Co_0.2_-NC-800 shows a better charging and discharging capacity (Fig. 6a), which is related to its ORR/OER performance [26, 34]. The generated discharge polarization and the corresponding power density are shown in Fig. 6b. The power density produced by Fe_8_Co_0.2_-NC-800 as air cathode is 124.9 mW cm^−2^, which is much higher than that of Pt/C + RuO_2_ (i.e., 103.9 mW cm^−2^). In order to evaluate the discharge process performance and battery capacity, the specific capacity of the battery at 3.5 mA cm^−2^ was tested. The results show that the specific capacity of the battery, which is used Fe_8_Co_0.2_-NC-800 as an air cathode, can reach as high as 704 mAh g_Zn_^−1^ (Fig. S24). The rate performance of the as-assembled battery was also tested through the constant current discharge test. As shown in Fig. 6c, the control current changed rapidly between 1–10 mA. In addition, the as-assembled battery has a stable discharge voltage platform under each tested current, and it could return to the original voltage platform when the current decreased. Such result suggests that Fe_8_Co_0.2_-NC-800 used as an air cathode has an excellent discharge rate performance. In terms of the constant current charge discharge test, Fe_8_Co_0.2_-NC-800 ZAB exhibited an excellent durability, where the stable charging and discharging platform can be maintained during a 311-h long-time operation (Fig. 6d) [40]. In the extended cycle voltage diagram (Fig. 6e), 4 cycles are randomly sampled in 1,701 cycles. It can be seen that the assembled ZAB could maintain stable charging and discharging performance at any time, although the battery discharge platform was attenuated due to the long-time cycle test in the last 50 h of test. In general, the round-trip efficiency of the battery can be maintained at 55.1%.

As flexible rechargeable ZAB has potentials in the field of wearable devices [45, 46], Fe_8_Co_0.2_-NC-800 was used as an air cathode to assemble the corresponding flexible ZAB devices, where zinc foil, alkaline polyacrylamide (PAM) and nickel foam was used as anode, flexible electrolyte and collector, respectively. The assembled Fe_8_Co_0.2_-NC-800 flexible ZABs has an open circuit voltage of 1.385 V and could remain stable within 400 s (Fig. 6f). Three connected ZABs in series can light an LED bulb with a rated voltage of 4 V (Fig. S25). Further test show that the flexible ZAB device has a power density of 6 mW cm^−2^ and is able to supply energy for some small devices (Fig. 6g-h).

### DFT Calculations

**Fig. 7:**
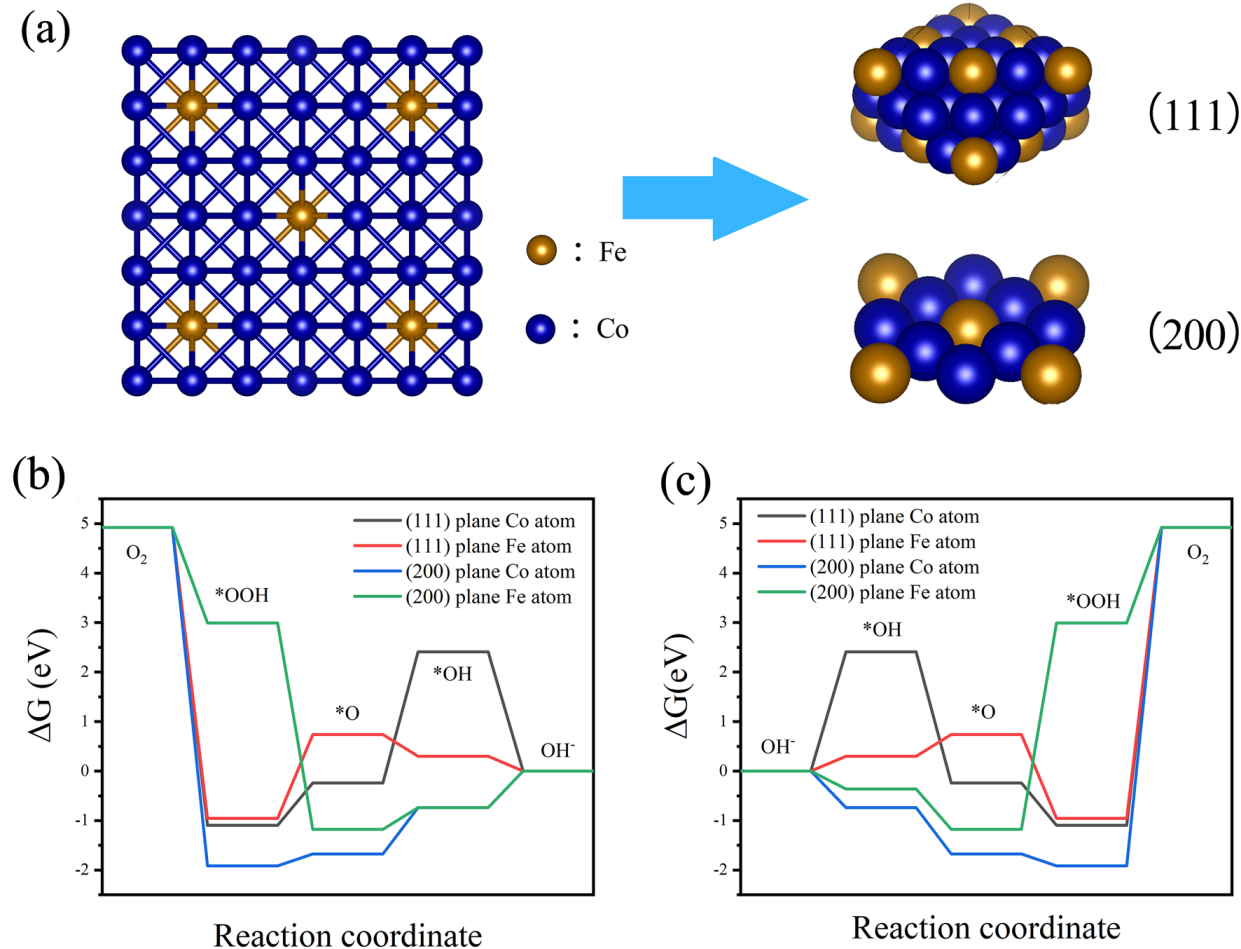
**a** Schematic diagram of Fe/Co NPs and (111), (200) active crystal plane structure; Calculated free-energy diagrams for **b** ORR process and **c** OER process

In order to further understand the high activity sites and catalytic mechanism of ORR and OER of Fe_8_Co_0.2_-NC-800, Fe/Co NPs were studied by DFT [47, 48]. Based on the characterization results of XRD, HRTEM and EXAFS, the exposed crystal planes of Fe/Co NPs include (111), (200) crystal planes. A model was then constructed. As shown in Fig. 7a, the blue ball and the yellow ball represent Co and Fe atoms respectively. We hope to find out the performance source and catalytic mechanism of the catalyst on the main exposed crystal planes. Therefore, according to the HRTEM and XRD characterization results, we cut out (111) crystal planes and (200) crystal planes on the model, and simulate the Co and Fe on the corresponding crystal planes respectively. The rate-determining step of the ORR reaction is the OH* formation for (111) Co, (200) Fe and (200) Co, while O* formation came to be rate-determining for (111) Fe (Fig. 7b). (111) Fe has higher catalytic activity [49]. Therefore, the highly dispersed Fe in Fe/Co NPs is more likely to become the active center, and the surrounding Co atoms play a strengthening role in the OH* step during the ORR process. In contrast, the simulation results of OER are different (Fig. 7c). As for the OER process, OOH* formation is the rate-determining step for all the surfaces except (200) Fe, which exhibits a rate-determining O* formation step. Fe can effectively improve the reaction rate and accelerate oxygen desorption, which is similar to the results reported in literature [50, 51]. Therefore, the samples prepared by Fe/Co co-doping show a superior ORR/OER activity, and a large number of exposed (111) crystal planes ensure a sufficient number of catalytic active sites. The above research confirms that the high dispersion of Fe in Fe/Co NPs plays an important role in strengthening the catalytic activity of the as-prepared Fe_8_Co_0.2_-NC-800 material.

## Conclusion

In summary, we proposed a dual regulation method to prepare a 3D honeycomb-like carbon-based catalyst co-doped with Fe/Co. The prepared Fe_8_Co_0.2_-NC-800 catalyst has the characteristics of highly dispersed Fe atoms, bowl-like carbon shell interconnection, and Fe/Co nanocrystal coating. Fe_8_Co_0.2_-NC-800 showed excellent ORR, OER and HER performances, where the ORR and OER performances are competitive to those of commercial noble metal-based catalysts. In addition, the ZAB assembled by Fe_8_Co_0.2_-NC-800 air cathode also exhibited excellent charging and discharging capacity and durability. It could maintain an excellent charging and discharging platform after a 311-h (1,701 cycles) long-term cyclic charging and discharging process. Based on the XAFS characterization and DFT calculation, the excellent performance of the as-prepared catalyst can be due to its large number of exposed (111) Fe/Co NPs, which provides a sufficient number of active sites. Besides, its special 3D honeycomb-like structure provided the carrier of active sites, thus enhancing the electron transport. This work provides a new idea to the design of cost-effective electrocatalysts for energy-related applications.

### Supplementary Information

Below is the link to the electronic supplementary material.

Supplementary file1 (PDF 4535 KB)
