# Supplementary material for: 3D Interconnected Honeycomb-Like Multifunctional Catalyst for Zn–Air Batteries
Source: Nanomicro Lett. 2022 Dec 31;15:26. doi: 10.1007/s40820-022-00959-6 (PMC9805485; doi:10.1007/s40820-022-00959-6)
Supplement: Supplementary file 1 — Supplementary file1 (PDF 4535 KB) [file 40820_2022_959_MOESM1_ESM.pdf]

Supporting Information for

3D Interconnected Honeycomb-Like Multifunctional Catalyst for Zn–Air Batteries

Tianxu Jin[1], Junli Nie[1] Mei Dong[1], Binling Chen[2,]*, Jun Nie[1], Guiping Ma[1,]*

[1]State Key Laboratory of Chemical Resource Engineering, Beijing University of Chemical Technology, Beijing 100029, P. R. China

[2]College of Engineering, Mathematics and Physical Science, University of Exeter, Exeter EX4 4QF, UK

*Corresponding authors. E-mail: magp@mail.buct.edu.cn (Guiping Ma), b.chen@exeter.ac.uk (Binling Chen)

Supplementary Figures and Tables

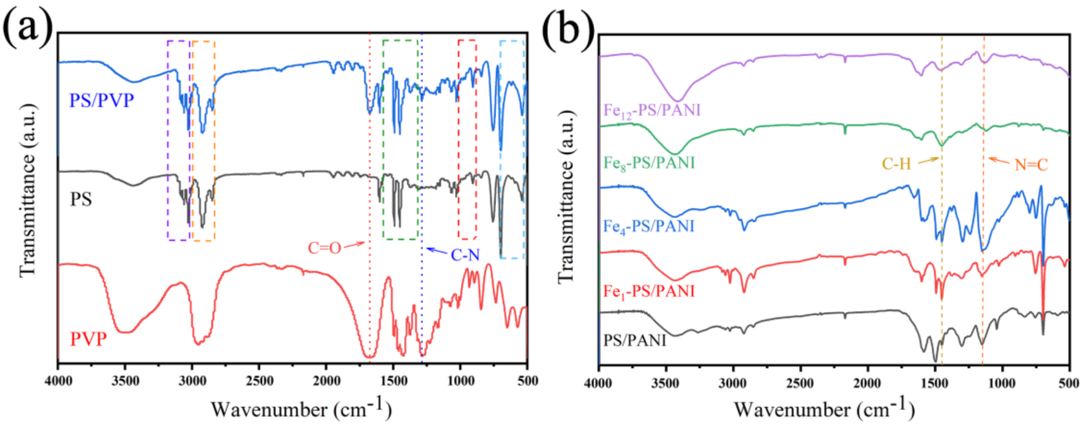

Fig. S1 (**a**) FTIR spectrum of pure PS, pure PVP and PS / PVP composite samples; (**b**) FTIR spectrum of PS / PANI, Fe_1-PS / PANI, Fe_4-PS / PANI, Fe_8-PS / PANI and Fe_{12}-PS / PANI

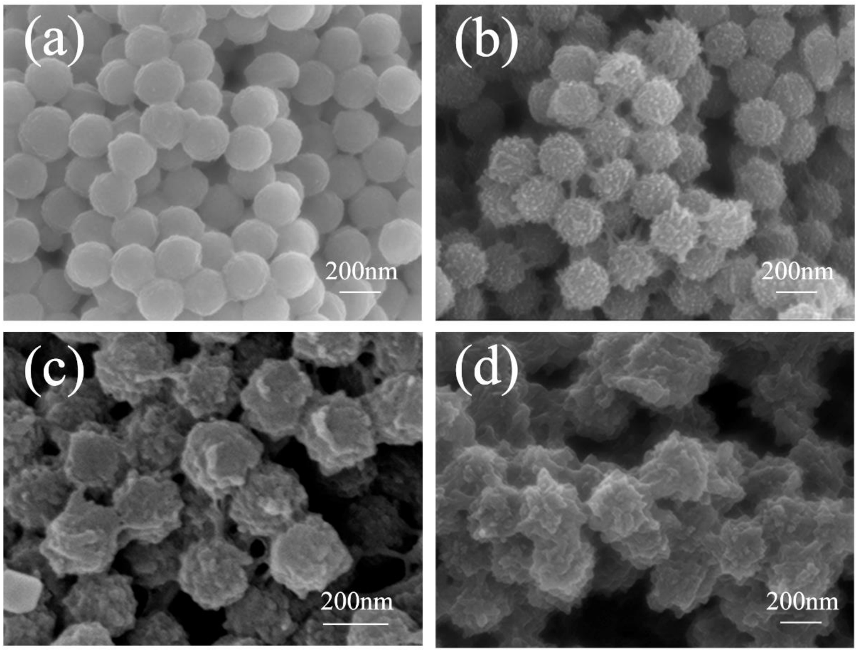

Fig. S2 SEM images of (**a**) Fe_1-PS / PANI; (**b**) Fe_4-PS / PANI; (**c**) Fe_8-PS / PANI and (**d**) Fe_{12}-PS / PANI

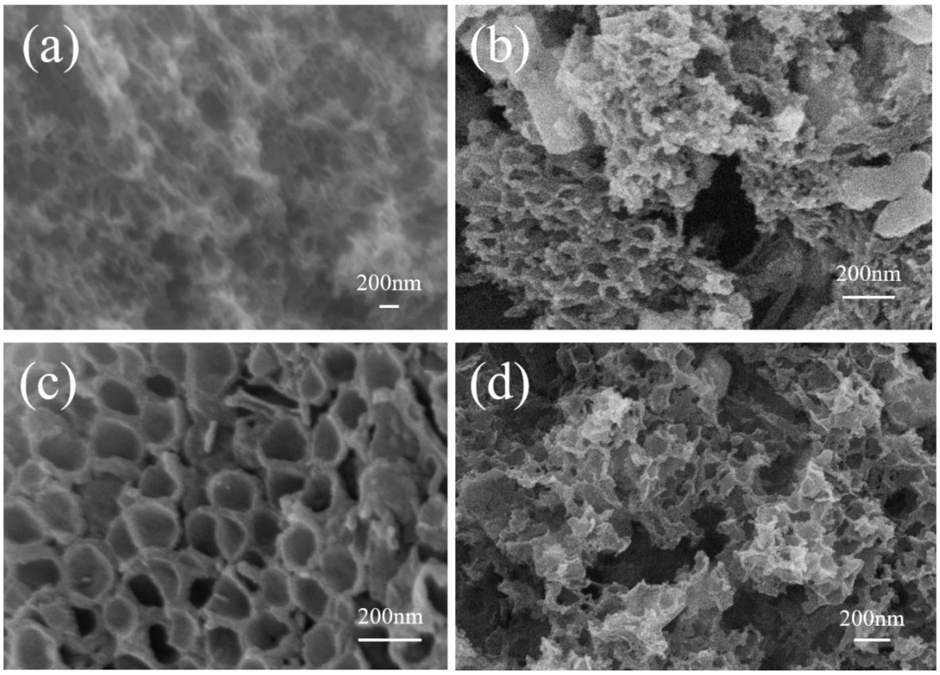

Fig. S3 SEM images of (**a**) Fe_1-NC-800; (**b**) Fe_4-NC-800; (**c**) Fe_8-NC-800 and (**d**) Fe_{12}-NC-800

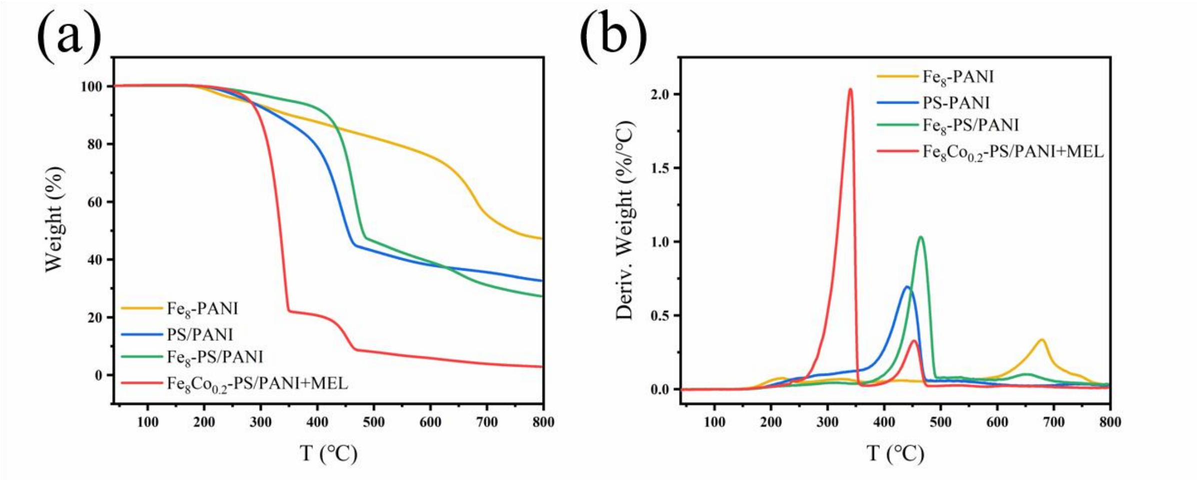

Fig. S4 SEM images of (**a**) Fe_1-PS/PANI; (**b**) Fe_4-PS/PANI; (**c**) Fe_8-PS/PANI and (**d**) Fe_{12}-PS/PANI

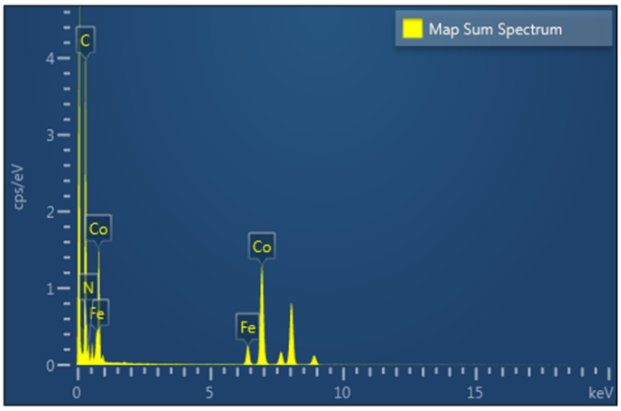

Element	Wt%	Wt% Sigma	Atomic %
C	64.92	0.29	86.58
N	4.37	0.27	5.00
Fe	4.56	0.09	1.31
Co	26.14	0.21	7.11
Total:	100.00		100.00

Fig. S5 the contents of different elements of $Fe_8Co_{0.2}$-NC-800 were measured by energy dispersive X-ray spectroscopy (EDS)

Table S1 ICP-MS of Fe and Co from $Fe_8Co_{0.2}$-NC-800

Sample	Element	Content (ppm)
$Fe_8Co_{0.2}$-NC-800	Fe	0.2299
	Co	0.3150

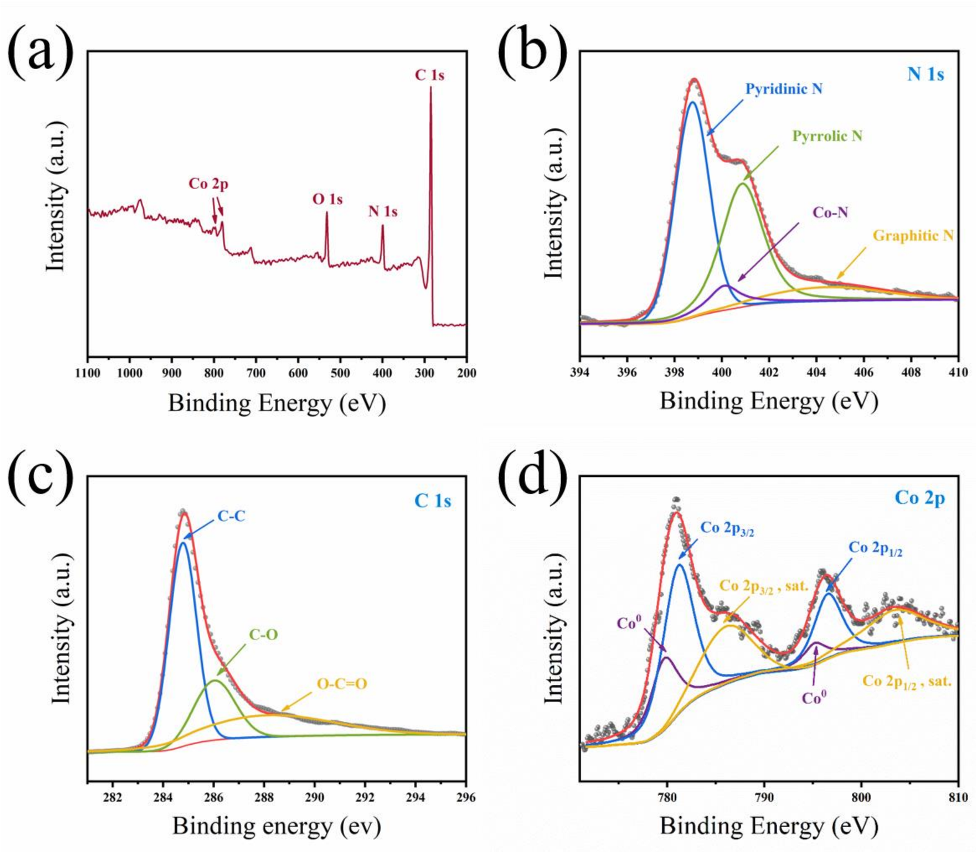

Fig. S6 (**a**) XPS full spectrum of $Co_{0.2}$-NC-800; High resolution spectra of (**b**) N 1s, (**c**) C 1s, (**d**) Co 2p

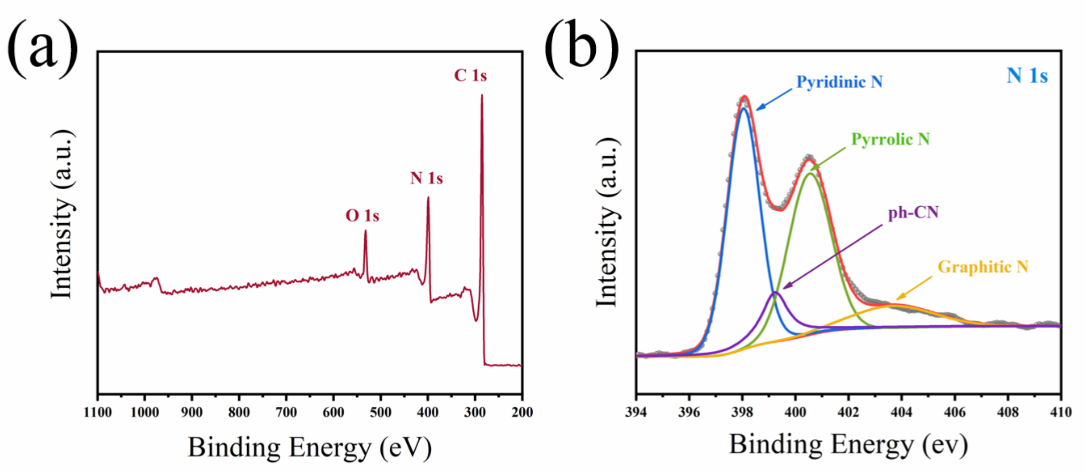

Fig. S7 (**a**) XPS full spectrum of NC-800; High resolution spectra of (**b**) N 1s

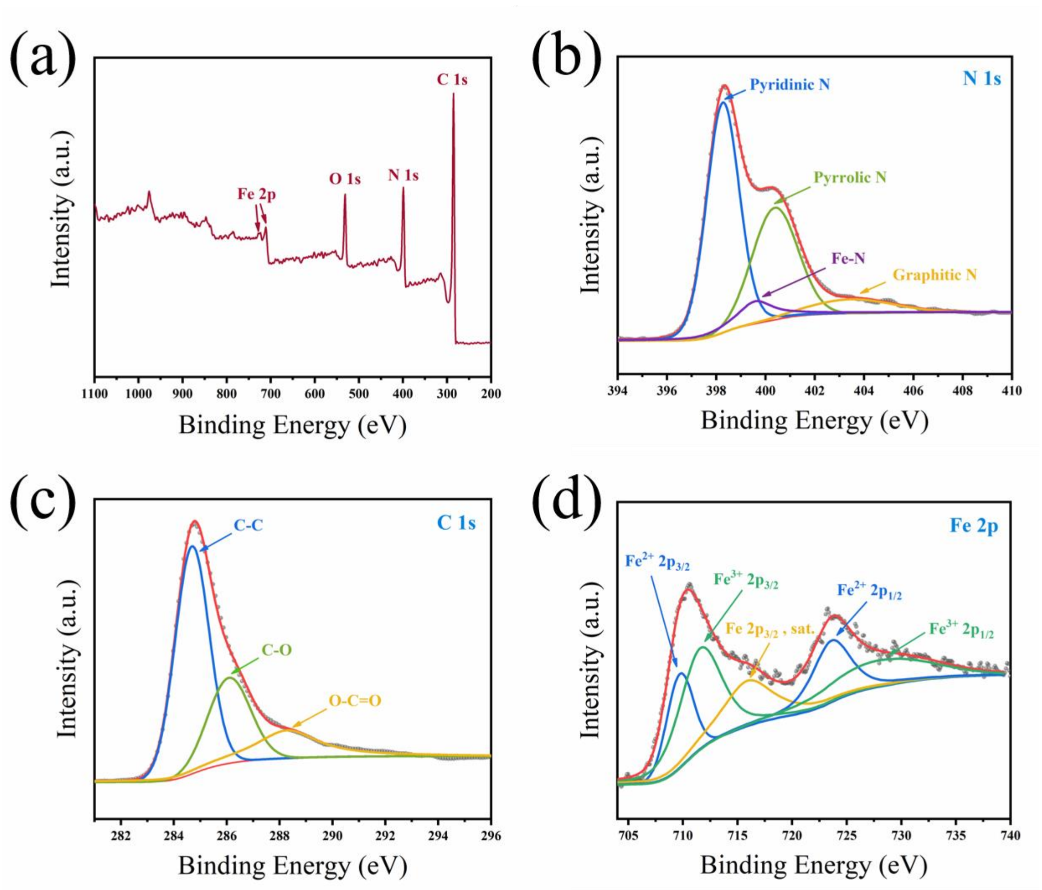

Fig. S8 (**a**) XPS full spectrum of $Fe_8Co_{0.2}$-NC-800; High resolution spectra of (**b**) N 1s, (**c**) C 1s, (**d**) Fe 2p

Table S2 EXAFS fitting parameters at the Co K-edge for various samples (S_0^2=0.791)

Sample	Shell	CN^a	R(Å)^b	σ^2(Å^2)^c	ΔE_0(eV)^d	*R* factor
Co foil	Co-Co	12*	2.49±0.01	0.0061±0.0002	7.1±0.4	0.002
CoPc	Co-N	4.0±1.0	1.89±0.01	0.0025±0.002	3.48±3.2	0.03
CoO	Co-O	6.0±0.9	2.12±0.01	0.01±0.0041	0.16±1.8	0.004
	Co-Co	12.6±1.2	3.00±0.01	0.009±0.0010	-2.46±0.9	
Sample Co	Co-N	3.0±2.1	2.0±0.01	0.013±0.014	3.19±9.89	0.004
	Co-Fe/Co	6.0±0.6	2.5±0.01	0.004±0.0008	-3.43±1.34	

Table S3 EXAFS fitting parameters at the Fe K-edge for various samples (S_0^2=0.59)

Sample	Shell	CN^a	R(Å)^b	σ^2(Å^2)^c	ΔE_0(eV)^d	*R* factor
Fe foil	Fe-Fe1	8*	2.46±0.01	0.004±0.0001	-2.77±1.68	0.007
	Fe-Fe2	6*	2.84±0.01	0.004±0.0001	-3.65±3.11	
FePc	Fe-N	3.9±0.67	1.95±0.01	0.007±0.002	-0.9±2.4	0.016
Fe3O4	Fe-O	6.3±1.6	1.93±0.01	0.012±0.004	-7.0±3.8	0.02
	Fe-Fe	12.8±3.8	3.09±0.01	0.016±0.003	6.5±2.4	
Sample Fe	Fe-N	3.1±1.3	1.98±0.01	0.011±0.006	-0.71±5.54	0.009
	Fe-Fe/Co	5.0±0.9	2.52±0.01	0.005±0.0018	-2.18±2.55	

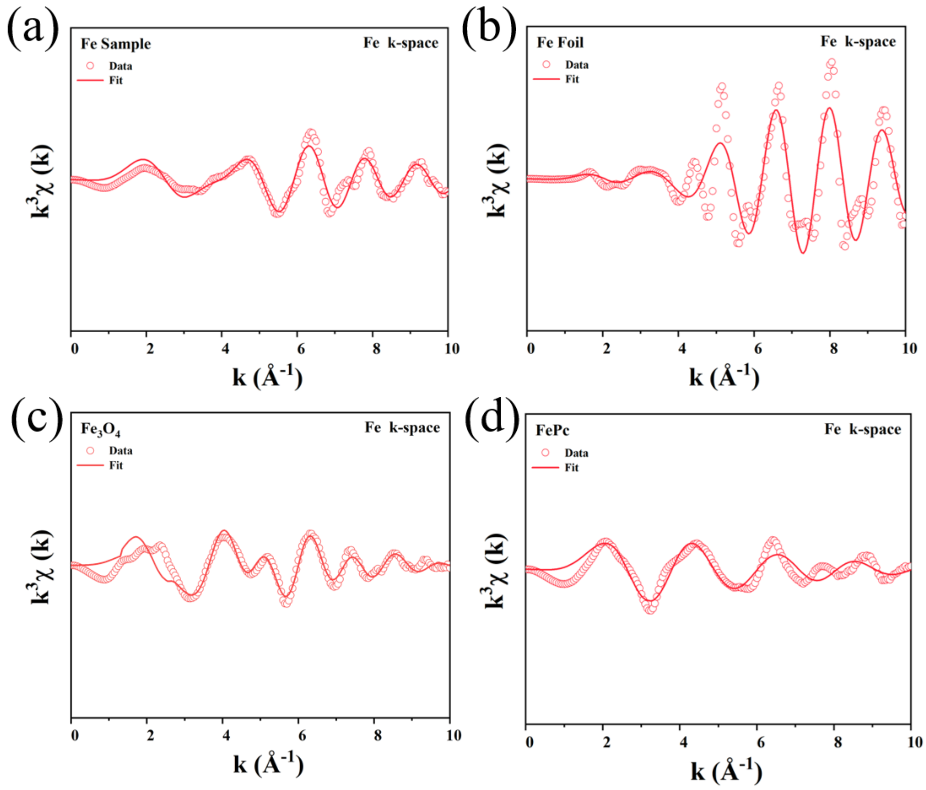

Fig. S9 EXAFS fitting curve of Fe element sample in k-space

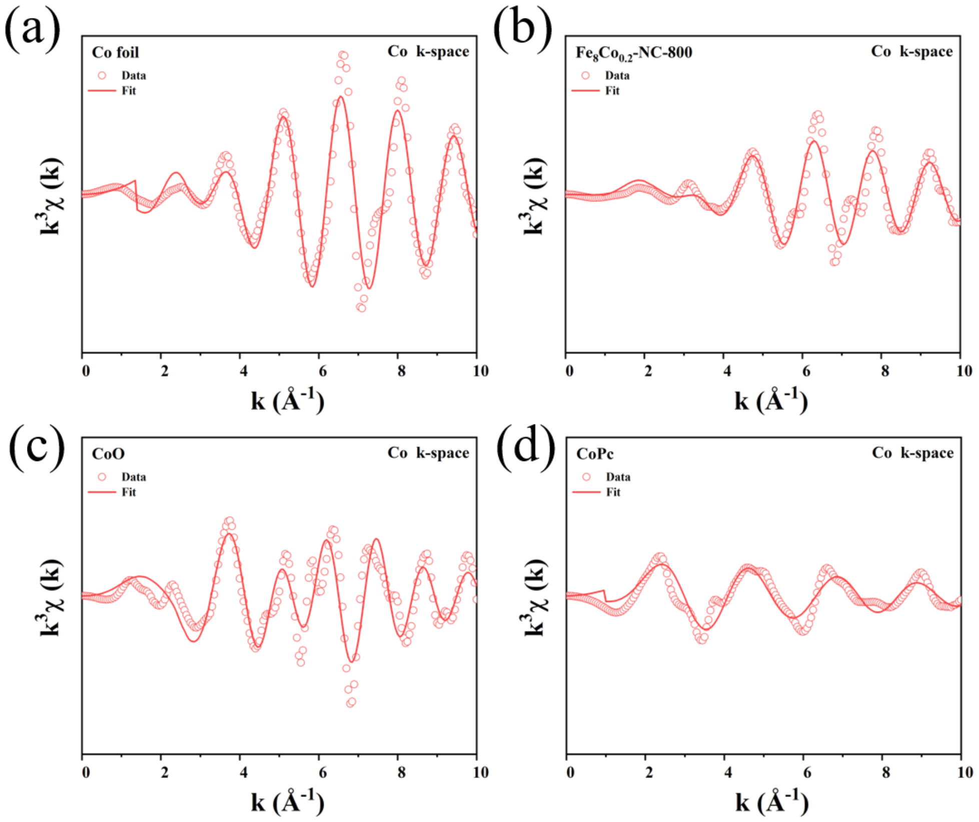

Fig. S10 EXAFS fitting curve of Co element sample in k-space

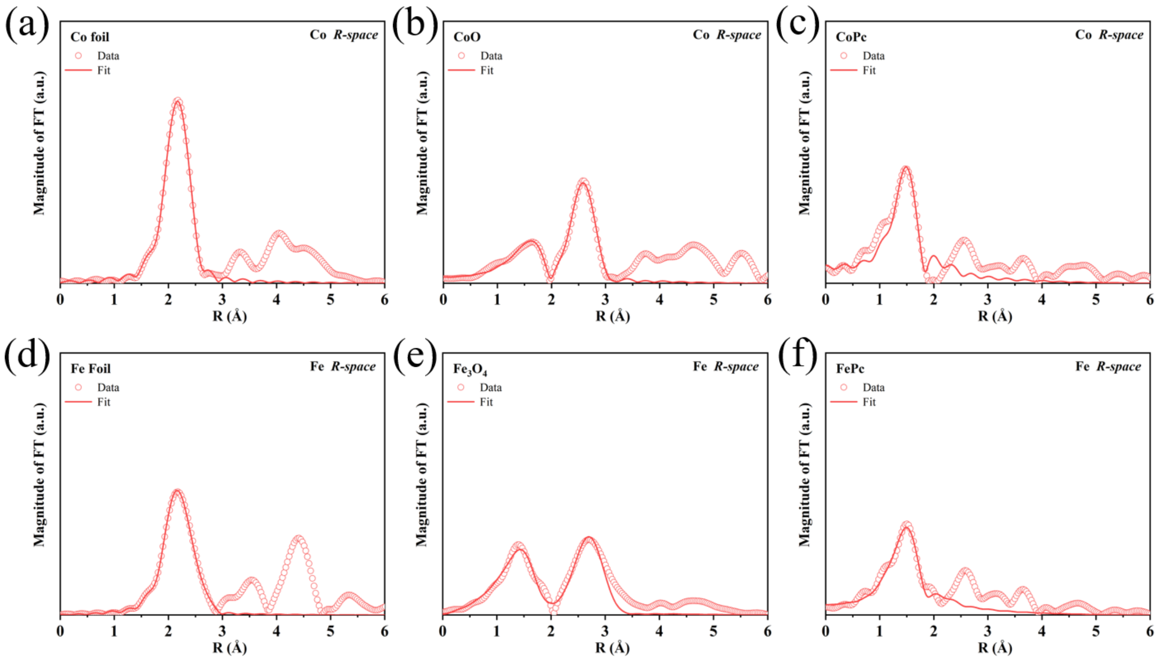

Fig. S11 EXAFS fitting curve of reference samples of Co and Fe in R-space

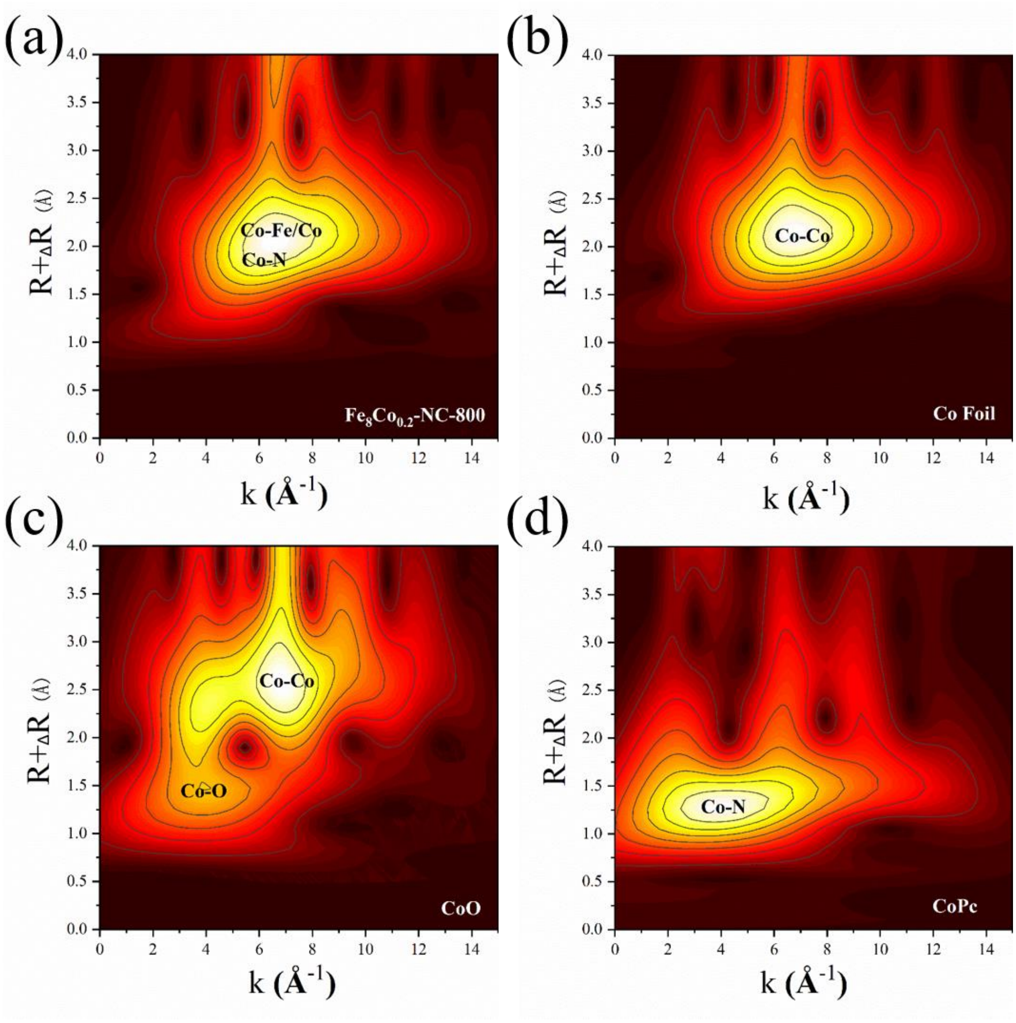

Fig. S12 Co K edge wavelet transform extended X-ray absorption fine structure (WT-EXAFS) of (**a**) $Fe_8Co_{0.2}$-NC-800, (**b**) Co Foil, (**c**) CoO, (**d**) CoPc

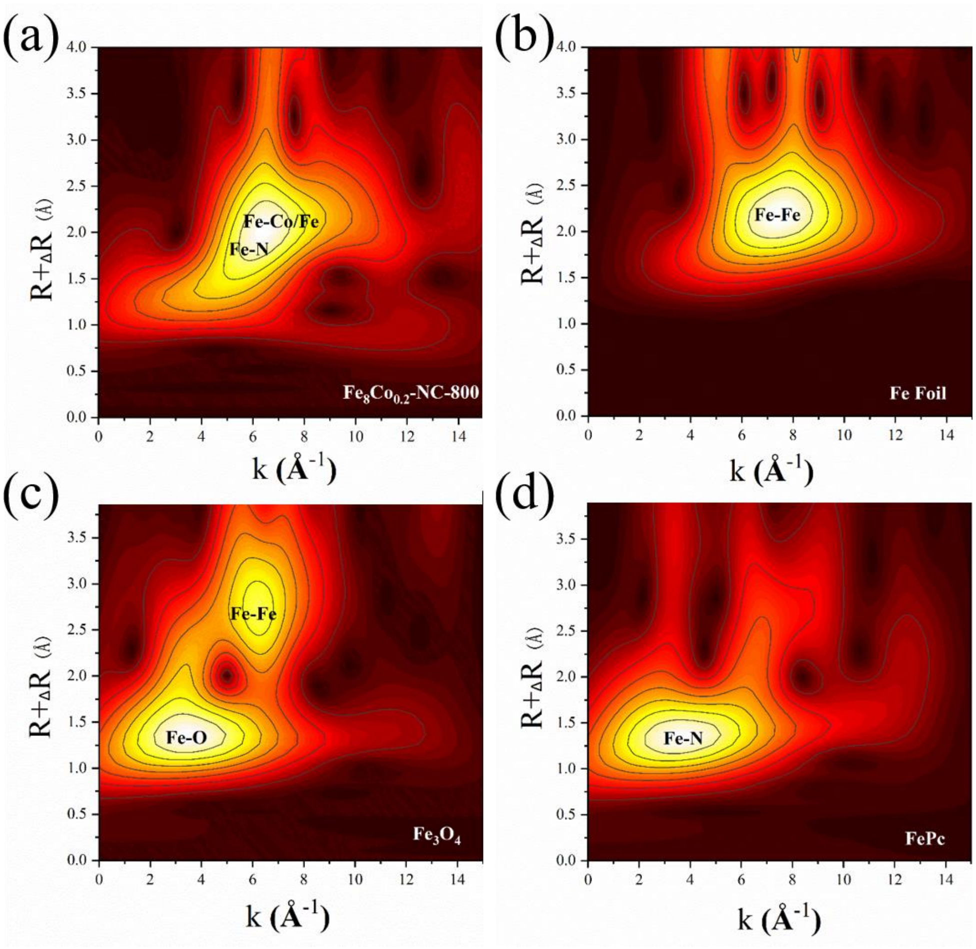

Fig. S13 Fe K edge wavelet transform extended X-ray absorption fine structure (WT-EXAFS) of (**a**) $Fe_8Co_{0.2}$-NC-800, (**b**) Fe Foil, (**c**) Fe_3O_4, (**d**) FePc

For Wavelet Transform analysis, the χ(k) exported from Athena was imported into the Hama Fortran code. The parameters were listed as follow: R range, 1 - 4 Å, k range, 0 - 15.0 Å^{-1} for **Co**、**Fe** sample; k weight, 2; and Morlet function with κ=5, σ=1 was used as the mother wavelet to provide the overall distribution.

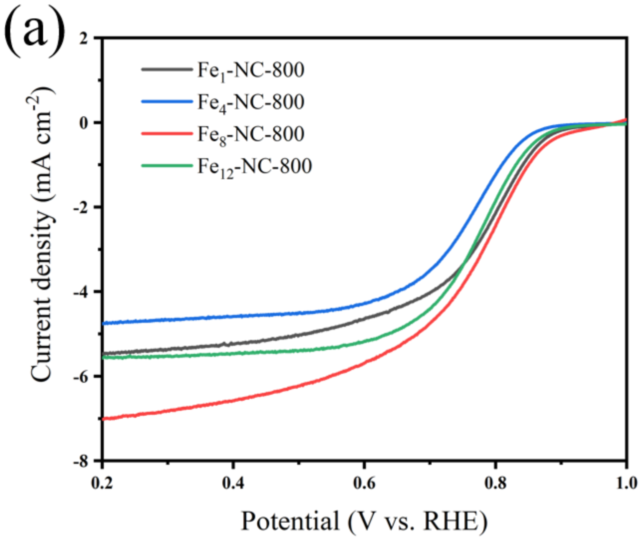

Fig. S14 LSV curve of ORR of Fe_x-NC-800 catalysts

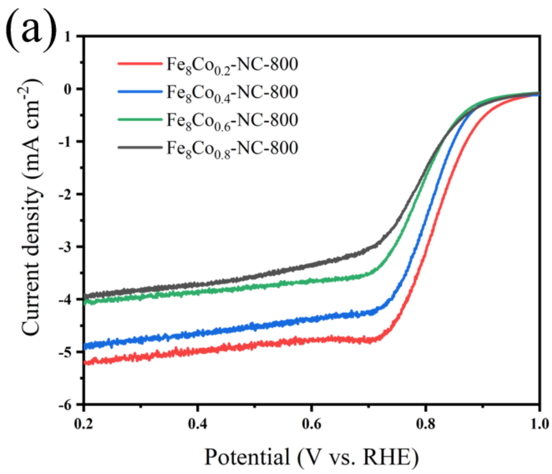

Fig. S15 LSV curve of ORR of catalysts with different Co content

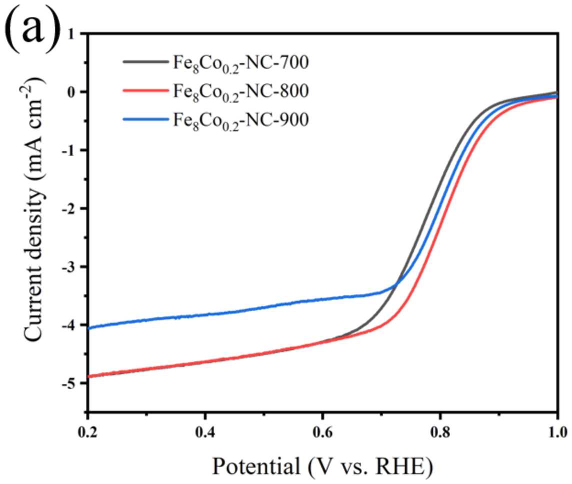

Fig. S16 LSV curves of ORR of catalysts prepared at different carbonization temperatures

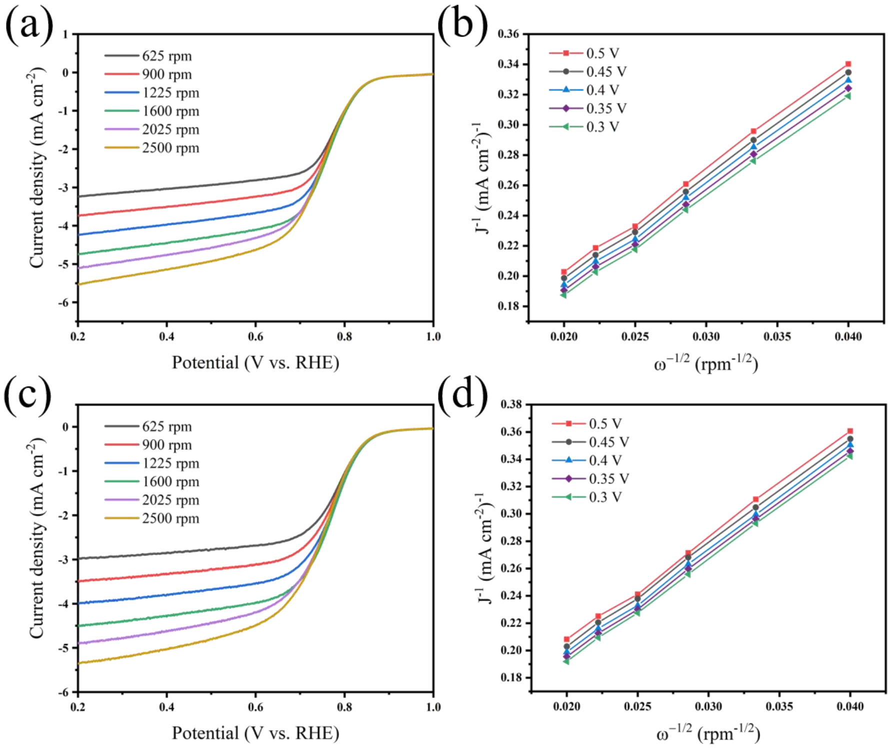

Fig. S17 (**a, c**) LSV curve of $Fe_8Co_{0.2}$-NC-700, $Fe_8Co_{0.2}$-NC-900 at different rotating speeds and corresponding (**b, d**) K-L curve

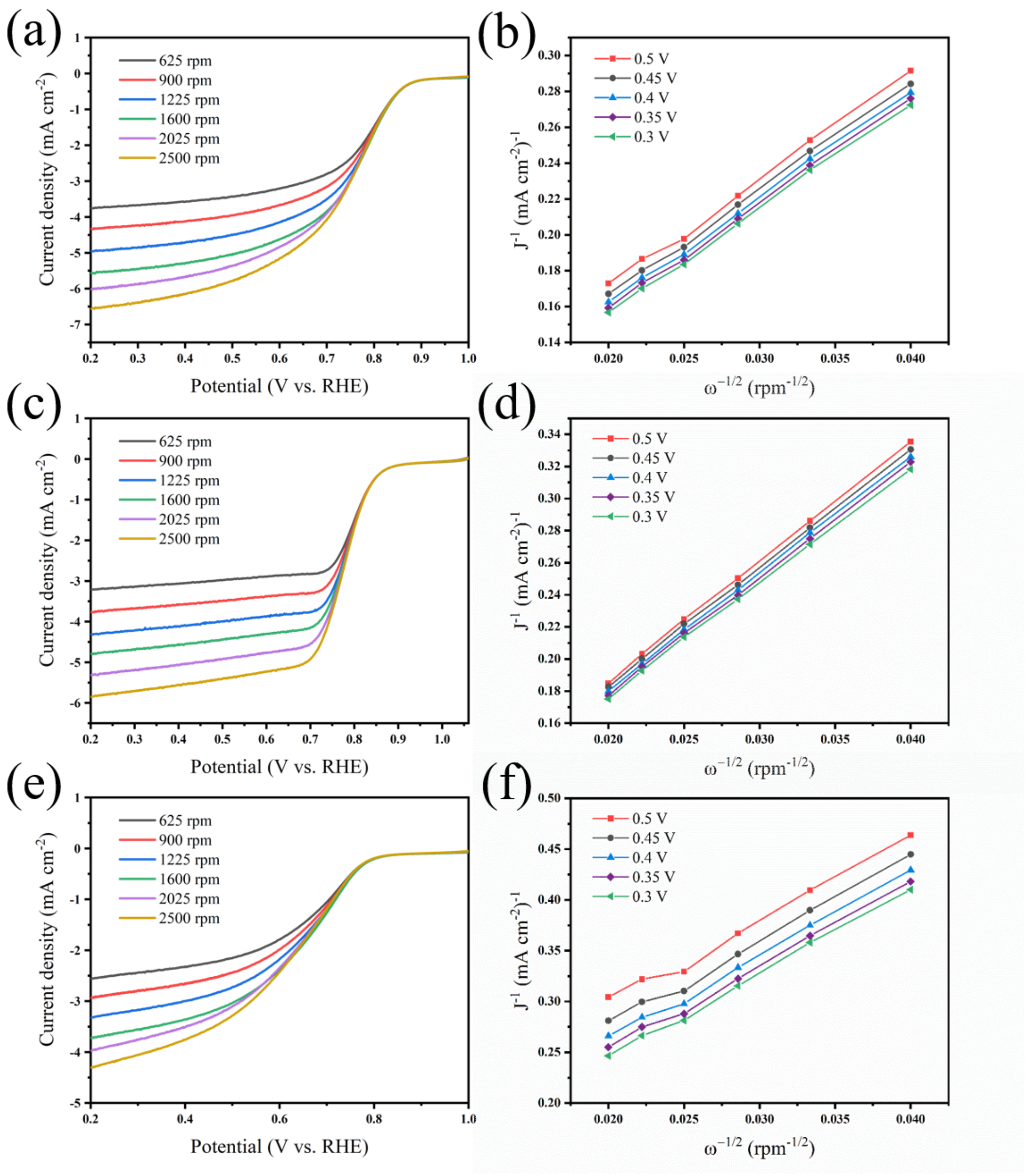

Fig. S18 LSV curve of Fe_8-NC-800、$Co_{0.2}$-NC-800、NC-800 at different rotating speeds and corresponding K-L curve

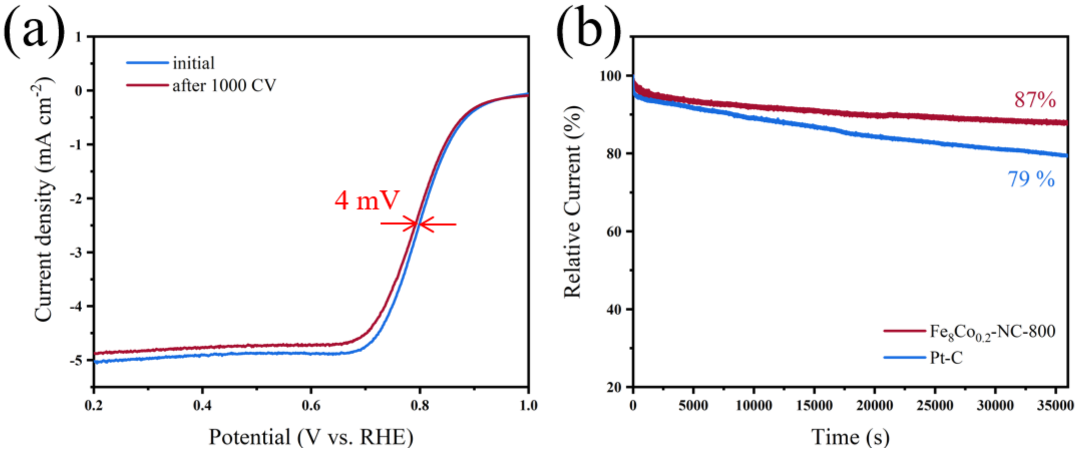

Fig. S19 ORR catalytic durability test of $Fe_8Co_{0.2}$-NC-800

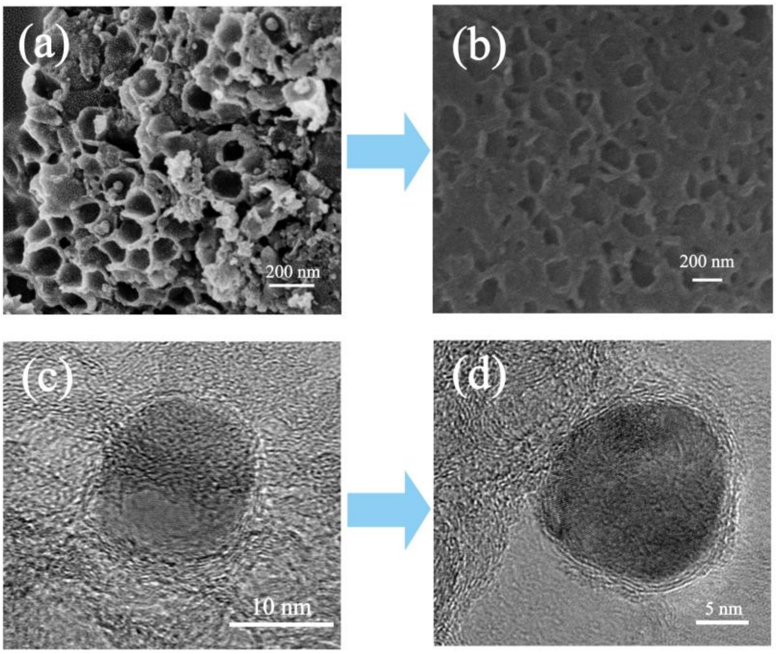

Fig. S20 Morphological changes of $Fe_8Co_{0.2}$-NC-800 before and after durability test. (**a-b**) SEM, (**c-d**) TEM

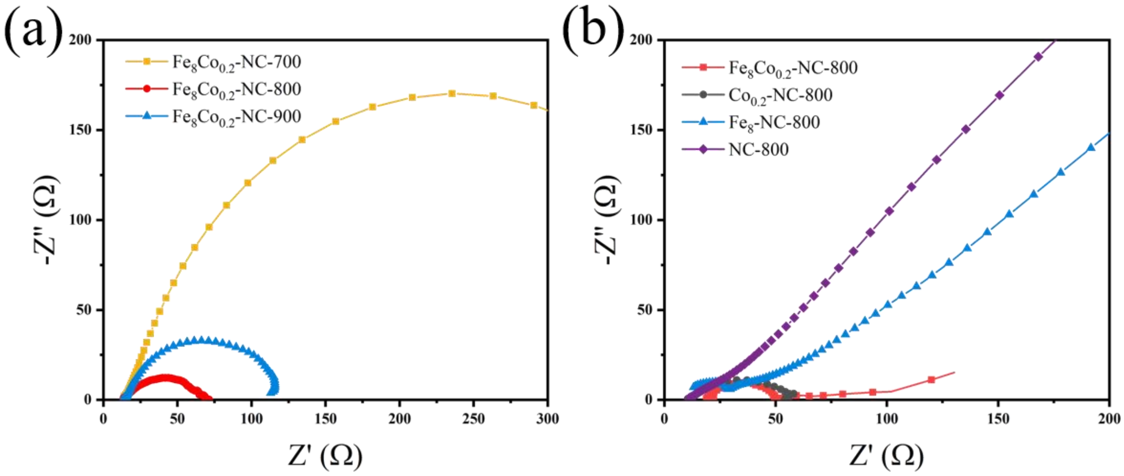

Fig. S21 EIS spectra of the prepared samples

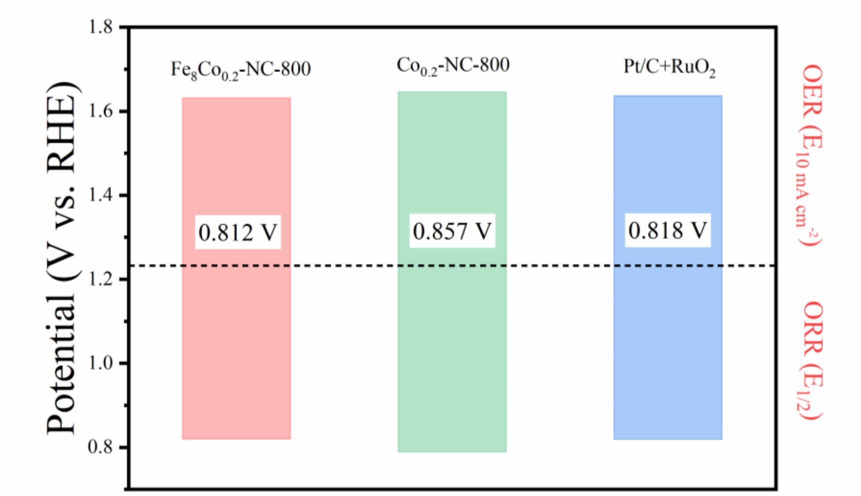

Fig. S22 Voltage difference (ΔE) statistical chart of as-prepared catalysts for OER/ORR catalytic activity

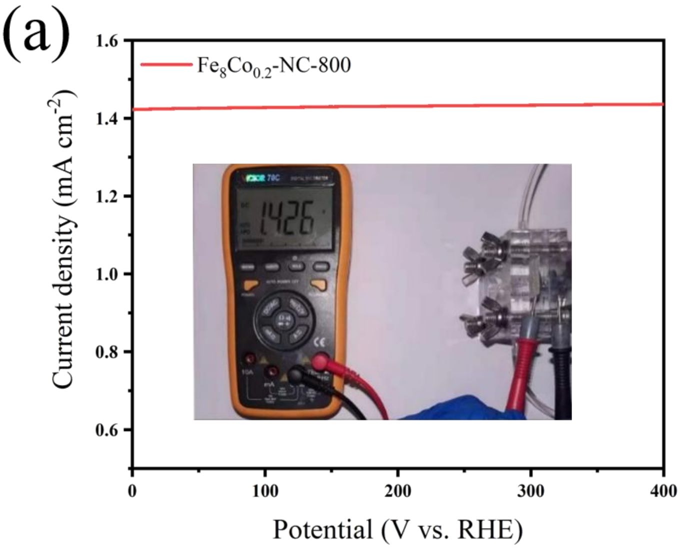

Fig. S23 Open circuit voltage test of liquid battery (inset is digital photo of multimeter test)

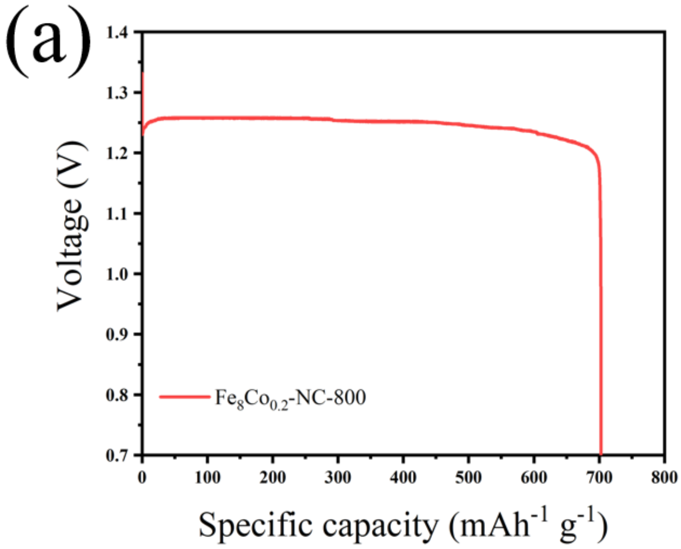

Fig. S24 Constant current discharge curve of liquid battery at 3.5 mA cm^{-2} and the specific capacity are normalized by Zn mass

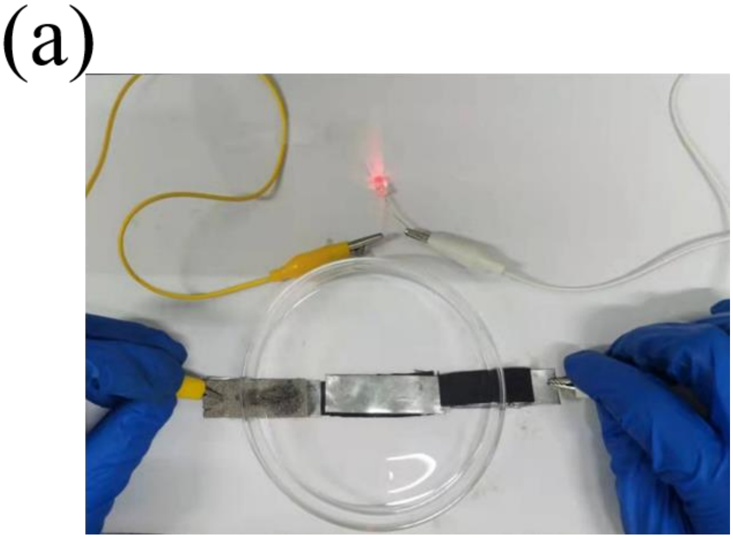

Fig. S25 Three batteries in series can light one LED bulb

Table S4 Calculated ORR free energy

Configuration	Reaction free energy (eV)				
	$2H_2O+O_2$	OH^- $+H_2O+OOH^*$	$2OH^-$ $+H_2O+O^*$	$3OH^-+OH^*$	$4OH^-$
111Co	4.92	-1.0937	-0.2416	2.4099	0
111Fe	4.92	-0.9554	0.7368	0.3002	0
200Co	4.92	-1.9136	-1.6742	-0.7388	0
200Fe	4.92	2.9916	-1.1786	-0.3631	0

Table S5 Calculated OER free energy

Configuration	Reaction free energy (eV)				
	$2H_2O+O_2$	OH^- $+H_2O+OOH^*$	$2OH^-$ $+H_2O+O^*$	$3OH^-+OH^*$	$4OH^-$
111Co	0	6.0137	5.1616	2.5101	4.92
111Fe	0	5.8754	4.1832	4.6197	4.92
200Co	0	6.8336	6.5942	5.6588	4.92
200Fe	0	1.9283	6.0986	5.2831	4.92

Table S6 Performance comparison with the catalyst reported in the literature

**Material**	**ORR $E_{1/2}$ [V vs RHE]**	**OER E_{10} [V vs RHE]**	**HER E_{10} [V vs RHE]**	**Refs.**
$Fe_8Co_{0.2}$-NC-800	0.82 V	1.63 V	0.29 V	This Work
NiFe-LDH/NrGO	0.75 V	1.47 V	/	[S1]
CoDNi-N/C	0.81 V	1.59 V	/	[S2]
FeCo@C MS	0.85 V	1.67 V	0.22 V (acid)	[S3]
Fe-Co(0.4)/N-rGO-AL	0.81 V	/	/	[S4]
Fe/ Co-CNT@MXene-8	0.85 V	1.61 V	/	[S5]
N-Co_3O_4@NC	0.77 V	1.50 V	/	[S6]
CC-AC	0.75 V	1.60 V	/	[S7]
1-NH_2	0.76V	1.45 V	/	[S8]
Ca_2FeRuO_6	0.8 V	1.57 V	0.19 V	[S9]
NCF-900	0.89 V	1.57 V	0.20 V (NRR)	[S10]

Supplementary References

[S1] T. Zhan, X. Liu, S. Lu, W. Hou, Nitrogen doped NiFe layered double hydroxide/reduced graphene oxide mesoporous nanosphere as an effective bifunctional electrocatalyst for oxygen reduction and evolution reactions. Appl. Catal. B Environ. **205**, 551-558 (2017). http://dx.doi.org/10.1016/j.apcatb.2017.01.010

[S2] Z. Li, H. He, H. Cao, S. Sun, W. Diao et al., Atomic Co/Ni dual sites and Co/Ni alloy nanoparticles in N-doped porous Janus-like carbon frameworks for bifunctional oxygen electrocatalysis. Appl. Catal. B Environ. **240**, 112-121 (2019). https://doi.org/10.1016/j.apcatb.2018.08.074

[S3] Y. Xu, B. Chen, J. Nie, G. Ma, Reactive template-induced core-shell FeCo@C microspheres as multifunctional electrocatalysts for rechargeable zinc-air batteries. Nanoscale **10**(36), 17021-17029 (2018). https://doi.org/10.1039/C8NR02492H

[S4] Q. Niu, J. Guo, Y. Tang, X. Guo, J. Nie et al., Sandwich-type bimetal-organic frameworks/graphene oxide derived porous nanosheets doped Fe/Co-N active sites for oxygen reduction reaction. Electrochim. Acta **255**, 72-82 (2017). https://doi.org/10.1016/j.electacta.2017.09.125

[S5] C. Zhang, H. Dong, B. Chen, T. Jin, J. Nie et al.,3D MXene anchored carbon nanotube as bifunctional and durable oxygen catalysts for Zn–air batteries. Carbon **185**, 17-26 (2019). https://doi.org/10.1016/j.carbon.2021.09.004

[S6] Z. Wang, W. Xu, X. Chen, Y. Peng, Y. Song et al., Defect-rich nitrogen doped Co_3O_4 /C porous nanocubes enable high-efficiency bifunctional oxygen electrocatalysis. Adv. Funct. Mater. **29**(33), 1902975 (2019). https://doi.org/10.1002/adfm.201902875

[S7] K. Kordek, L. Jiang, K. Fan, Z. Zhu, L. Xu et al., Two-step activated carbon cloth with oxygen-rich functional groups as a high-performance additive-free air electrode for flexible zinc–air batteries. Adv. Energy Mater. **9**(4), 1802936 (2018). https://doi.org/10.1002/aenm.201802936

[S8] W. Li, S. Xue, S. Watzele, S. Hou, J. Fichtner et al., Advanced bifunctional oxygen reduction and evolution electrocatalyst derived from surface-mounted metal-organic frameworks. Angew. Chem. Int. Ed. **59**(14), 5837-5843 (2020). https://doi.org/10.1002/anie.201916507

[S9] N. Kumar, K. Naveen, M. Kumar, T.C. Nagaiah, R. Sakla et al., Multifunctionality exploration of Ca_2FeRuO_6: an efficient trifunctional electrocatalyst toward OER/ORR/HER and photocatalyst for water splitting. ACS Appl. Energy Mater. **4**(2), 1323-1334 (2021). https://dx.doi.org/10.1021/acsaem.0c02579

[S10] X. Yang, K. Li, D. Cheng, W.L. Pang, J. Lv et al., Nitrogen-doped porous carbon: highly efficient trifunctional electrocatalyst for oxygen reversible catalysis and nitrogen reduction reaction. J. Mater. Chem. A **6**(17), 7762-7769 (2018). https://doi.org/10.1039/C8TA01078A
